# Protective Role of Matrix Metalloproteinase-2 in Allergic Bronchial Asthma

**DOI:** 10.3389/fimmu.2019.01795

**Published:** 2019-08-02

**Authors:** Yoshinori Takahashi, Tetsu Kobayashi, Corina N. D'Alessandro-Gabazza, Masaaki Toda, Kentaro Fujiwara, Tomohito Okano, Hajime Fujimoto, Kentaro Asayama, Atsuro Takeshita, Taro Yasuma, Kota Nishihama, Ryo Inoue, Liqiang Qin, Yoshiyuki Takei, Osamu Taguchi, Esteban C. Gabazza

**Affiliations:** ^1^Department of Pulmonary and Critical Care Medicine, Mie University Graduate School of Medicine, Tsu, Japan; ^2^Department of Immunology, Mie University Graduate School of Medicine, Tsu, Japan; ^3^Department of Diabetes, Metabolism and Endocrinology, Mie University Graduate School of Medicine, Tsu, Japan; ^4^Central Institute for Experimental Animals, Kawasaki-ku, Japan; ^5^Department of Nephrology, Taizhou Hospital, Wenzhou Medical University, Lihai, China; ^6^Center for Physical and Mental Health, Mie University Graduate School of Medicine, Tsu, Japan

**Keywords:** matrix metalloproteinases, mouse models, bronchial asthma, nitric oxide, Th2 cytokines

## Abstract

Inflammation, reversible obstruction, and hyperresponsiveness of the airways are characteristic findings of bronchial asthma. Several evidence has demonstrated the involvement of matrix metalloproteinase-2 in allergic airway inflammation. Matrix metalloproteinase-2 may promote aberrant tissue remodeling in late stages of allergic airway inflammation. However, whether matrix metalloproteinase-2 is detrimental or protective in early stages of allergic airway inflammation remains unclear. To evaluate this here we compared the severity of allergic bronchial asthma between mice overexpressing human matrix metalloproteinase-2 and wild type mice. After sensitization and challenge with an allergen, mice overexpressing the human matrix metalloproteinase-2 showed a significant reduction in airway hyperresponsiveness and in the expression of Th2 cytokines and IgE compared to their wild type counterparts. An inhibitor of matrix metalloproteinases abolished this beneficial effect of human matrix metalloproteinase-2 overexpression. Allergen-sensitized and challenged human matrix metalloproteinase-2 transgenic mice had enhanced percentage of M1 macrophages with increased expression of inducible nitric oxide synthase and STAT1 activation in the lungs compared to their wild type counterparts. There was no difference in the percentage of regulatory T cells between mouse groups. The results of this study showed that matrix metalloproteinase-2 is protective in allergic bronchial asthma by promoting polarization of macrophages to M1 phenotype.

## Introduction

Bronchial asthma represents a serious public health problem with more than 330 million people affected worldwide and ~250,000 patients per year dying for the disease (1–4). Asthma is also a disabling illness (4). It affects the quality of life of the patient and family members, productivity at work, performance at school and use of health care insurance (4). The economic burden of asthma is also substantial (5). A recent survey disclosed an estimated medical cost of 4 billion dollars in the year 1999 in Japan, and about 59 billion dollars in United States in 2007 (5, 6). Genetic and environment factors are involved in the pathogenesis of asthma. A high type two immune response and increased eosinophilic infiltration in overresponsive airways are the predominant features in the acute phase of the disease (7, 8). Excessive deposition of extracellular matrix components (collagen, periostin, tenascin) or tissue remodeling with narrowing and impaired elasticity of the airways predominate in the chronic stage (7, 8). Matrix metalloproteinases are the main regulators of tissue remodeling in the airways (9).

Matrix metalloproteinases belong to a large family of calcium-dependent and zinc-containing endopeptidases that play a critical role in the turnover and degradation of extracellular matrix proteins (10). In addition to modulating the components of the extracellular connective tissue, matrix metalloproteinases also regulate cell proliferation, differentiation, migration, apoptosis, and vessel regeneration indirectly by cleaving and activating vital molecules that control cell function, or directly by binding to cell surface molecules that trigger activation of intracellular pathways (10, 11). The present study focused on the role of matrix metalloproteinase-2 (MMP-2) or gelatinase A in bronchial asthma (9). Several evidence supports the role of MMP-2 in allergic inflammation of the airways (12–18). Excessive activity of MMP-2 and other metalloproteinases including MMP-9 may be detrimental in late stages of allergic airway inflammation by promoting aberrant tissue remodeling of the bronchial walls (13–16, 18). However, whether the expression of MMP-2 is detrimental or protective in early stages of allergic airway inflammation is unclear. There are studies showing high, low or unchanged expression level of MMP-2 in asthmatic conditions compared to control (19–25).

We conducted this study to gain insights into the role of MMP-2 in the early stages of allergic airway inflammation. For this purpose, we compared the severity of allergic bronchial asthma between transgenic mice overexpressing the human MMP-2 and wild type mice.

## Subjects, Materials, and Methods

### Patients

This study comprised 27 patients with bronchial asthma that consulted the Department of Pulmonary and Critical Care Medicine of Mie University Hospital. Based on the Global Initiative for Asthma (GINA) classification of asthma severity, there were four patients in level 2, 17 in level 3 and 6 in level 4 (26). Supplementary Table 1 describes the characteristics and treatment of the patients. The bronchial asthma of all patients were stable under therapy. All patients were receiving inhaled corticosteroids, and in addition 21 patients were receiving long-acting β adrenoceptor agonist, 13 leukotriene receptor antagonist, 7 theophylline, 3 oral prednisolone, and 1 long-acting muscarinic antagonist. The characteristics and therapeutic history of the patients were retrospectively obtained from medical records. IgE-specific allergens are described in Supplementary Table 1. Data obtained from age-matched 34 healthy subjects (mean age 59.7 ± 7.0 years-old; males, 13; females, 21) were used as controls. The institutional review board of Mie University approved the study protocol (Approval No 2846; February 19, 2015).

### Reagents

Chicken egg ovalbumin (OVA) and aluminum hydroxide were from Sigma (St. Louis, MO, USA). The IC-21 cell line was from the American Type Culture Collection (Manassas, VA), RPMI-1640 medium from Sigma-Aldrich (Saint Louis, MO) and fetal bovine serum (FBS) from Bio Whittaker (Walkersville, MD). Recombinant active MMP-2 was purchased from Calbiochemi Co (San Diego, CA).

### Animals

We have previously characterized the human MMP-2 transgenic (TG) mice in a C57BL/6 background (27). The protein structure and function of human MMP-2 are similar to its mouse counterpart; the human and mouse MMP-2 have similar cleavage ability of collagens, binding affinity to tissue inhibitor of metalloproteinase-2 molecular interaction with integrins (10). Wild-type (WT) littermates were the control mice. Female mice weighing 18–22 g and between 8 and 12 weeks old were used. All animals were in a pathogen-free environment, kept on a constant 12:12-h light–dark cycle in a temperature- and humidity-controlled room and given water and standard mouse food *ad libitum*. The Mie University's Committee on Animal Investigation approved the experimental protocol (Approval No 25-20/Hen1-Sai; September 12, 2015).

### Preparation of a Model of Bronchial Asthma With Allergic Inflammation

We prepared a model of bronchial asthma with allergic inflammation in early stages by sensitizing mice with ovalbumin and then challenged them with ovalbumin by inhalation. We sensitized mice by intraperitoneal (i.p.) injection of 10 μg ovalbumin (OVA) adsorbed with 2 mg aluminum hydroxide on day 0 followed by booster injections on days 7, 14, and 21. Non-sensitized animals received intraperitoneal injection of saline following the same schedule used for aluminum-precipitated OVA injections (Supplementary Figure 1). For OVA challenge, sensitized mice were exposed to aerosolized 2% OVA for 30 min/day on days 28, 29, and 30 (Supplementary Figure 1). We used the AZWELL UN-511 ultrasonic nebulizer (Osaka, Japan) for aerosol generation. Non-sensitized animals were exposed to aerosolized saline following the same schedule used for OVA inhalation described above.

### Experimental Design

There were four experimental groups (Supplementary Figure 1). WT mice receiving i.p. injection of OVA or saline and then OVA (WT/OVA group) or saline (WT/SAL group) by inhalation, and hMMP-2 TG mice similarly receiving i.p. injection of OVA or saline and subsequently OVA (hMMP-2 TG/OVA) or saline (hMMP-2 TG/SAL) by inhalation.

A separate experiment was performed to evaluate whether doxycycline, an inhibitor of matrix metalloproteinases, can block the effect of hMMP-2 overexpression. In this experiment, a group of hMMP-2 TG mice fed with baits containing 5% doxycycline and groups of WT and hMMP-2 TG mice fed with standard baits were sensitized and challenged with OVA or treated with saline following similar protocol described above.

Another independent experiment was performed to evaluate the percentage of M1 and M2 macrophages and CD4^+^CD25^+^ cells in lung tissues from mice with allergic bronchial asthma. In this experiment, hMMP-2 transgenic and WT mice were allocated in WT/SAL, WT/OVA, hMMP-2 TG/SAL, and hMMP-2 TG/OVA groups in a similar manner as described above.

### Bronchoalveolar Lavage Fluid (BALF) and Plasma Sampling

We euthanized all animals by intraperitoneal injection of high dose pentobarbital and collected samples for biochemical and histological examination. Plasma and BALF samples were collected as previously described (28). The BALF was centrifuged (1,000 g, 10 min, 4°C) and the cell-free supernatant was stored immediately at −80°C until use for biochemical analysis. The total number of cells in BALF was counted using a nucleocounter from ChemoMetec (Allerød, Denmark). For differential cell counting, BALF cells was centrifuged using a cytospin and stained with May–Grünwald–Giemsa (Merck, Darmstadt, Germany).

### Evaluation of T Cell Response in hMMP-2 TG Mice

Spleen cells were collected and analyzed *ex vivo* for OVA-specific T-cell activation and proliferation. We dissected the spleens with scissors into small pieces, incubated for 30 min at 37°C in 0.5 mg/ml collagenase solution, and then filtered using a mesh. Splenocytes were stained with 5 μM carboxyfluorescein succinimidyl ester (CFSE) and seeded in triplicate in 24-well culture plates at a density of 2 × 10^6^ cells/mL in RPMI-1640 medium supplemented with 10% FBS with or without 100 μg/mL OVA. Five μg/mL concanavalin A (ConA) was used as positive controls. After 48 h, cells were harvested and stained with phycoerythrin (PE)-conjugated anti-CD4^+^ Ab or PE-conjugated anti-CD8^+^ Ab (Biolegend, San Diego, CA) and analyzed by FACScan flow cytometer. Cytokines and immunoglobulins in culture supernatants were measured by enzyme immunoassays. Macrophages and T cells were characterized *in vivo* using PE/Cy5 anti-mouse F4/90 antibody, PE anti-mouse/human IL-5 antibody, Alexa fluor 488 anti-mouse CD197 (CCR7) antibody, and PE anti-mouse CD206 (MMR) antibody from Biolegend (San Diego, CA), and iNOS monoclonal antibody, Alexa fluor 488 from eBioscience (Santa Cruz, CA).

### Biochemical Analysis

Total protein was measured by dye-binding assay (Bio-Rad Laboratories, Hercules, CA). Allergen specific IgE in the blood of patients was detected by radioallergosorbent testing. IgE, IL-5, IL-4, IL-10, monocyte chemoattractant protein (MCP)-3 were measured using commercial immunoassay kits from BD Biosciences Pharmigen (San Diego, CA). IgG2c was measured using a commercial immunoassay kit from ThermoFisher Scientific (Vienna, Austria). MMP-2, MMP-9, tissue inhibitor of metalloproteinases-2 (TIMP2), IL-13, periostin, and eotaxin were measured using commercial enzyme immunoassay kits from R&D Systems (Minneapolis, MN). The kit used for measuring the plasma levela of MMP-2 in mice measure the active and pro-form of both mouse and human MMP-2 proteins. Nitrite ion in cell supernatant was measured using a Griess reagent kit purchased from Dojindo (Tokyo, Japan). Zymography of MMPs was performed as described (11).

### Histological Examination

After thoracotomy, the pulmonary circulation was flushed with saline before removing both lungs. The left lung of each mouse was perfused with 10% neutral buffered formalin and fixed in formalin for 24 h. After embedding in paraffin, the tissue sections were prepared, stained with hematoxylin & eosin (H&E) or periodic acid Schiff (PAS), and examined by light microscopy. The PAS-positive areas were counted using an Olympus BX50 microscope combined with an Olympus DP70 digital camera (Tokyo, Japan) using the Win ROOF image-processing software (Mitani Corp., Fukui, Japan) for Windows. An average of 10 high-magnification photos of random areas were taken from each mouse and the area positive for PAS stain was calculated.

### Measurement of Airway Hyperresponsiveness

Hyperresponsiveness to increasing concentrations of aerosolized methacholine was measured by unrestrained plethysmography using a whole-body plethysmograph system (Buxco Electronics, Sharon, CT) as previously described (29).

### Cell Culture and Stimulation With Active Human MMP-2

IC-21 macrophages were cultured in RPMI-1640 supplemented with 10% FBS in the presence of 10 ng/ml of active human MMP-2, and then after 1 h stimulated with 50 ng/ml of IFNγ or 10 ng/ml of IL-4 and cells were then collected after 24 h for RNA extraction.

### Western Blotting

We performed Western blotting of Akt, p-AKT, STAT1, p-STAT1, STAT6, p-STAT6, and β-actin as previously described using antibodies from Cell Signaling Technology (Danvers, MA) (27).

### RNA Extraction, Reverse Transcription Polymerase-Chain Reaction

Total RNA was extracted using the Sepasol RNA I super G (Nacalai), according to the manufacturer's instructions. The quantity and quality of resulting RNA was assessed using a Nano Drop ND-1000 spectrophotometer (Shimazu). A total of 200–500 ng of RNA per sample was reverse-transcribed to cDNA using oligo dT primers and ReverTraAce reverse transcriptase (TOYOBO), according to the manufacturer's instructions. Supplementary Table 2 shows the primers used in the experiments.

### Statistical Analysis

Data were expressed as the mean ± standard deviation of the mean (S.D.) unless otherwise specified. For the statistical test, we used analysis of variance along with the Tukey test for the *post hoc* analysis among four variables, and by Mann-Whitney *U*-test between two variables. The difference in sex distribution between patients and controls was analyzed by Fisher's exact test. Statistical analyses were done using the Graph Pad Prism package software for Windows (Graph Pad Software, Inc., La Jolla, CA). Statistical significance was considered as *p* < 0.05.

## Results

### Decreased Plasma Levels of MMP-2 in Asthmatic Patients

**Figure 1:**
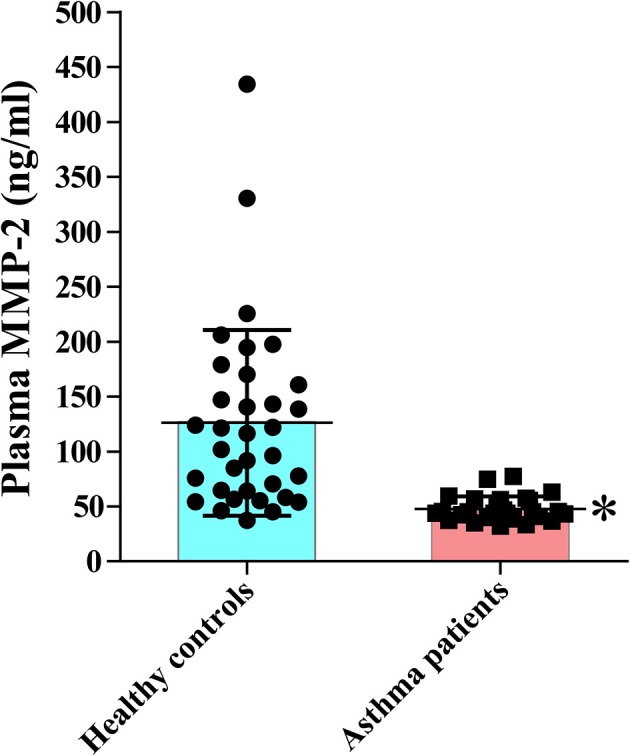
Reduced circulating MMP-2 in patients with bronchial asthma. The study comprised 27 patients with bronchial asthma and 34 healthy subjects. The levels of MMP-2 were measured using commercial immunoassay kits. Bars indicate the means ± S.D. Statistical difference was evaluated by Mann-Whitney *U*-test. MMP-2, human matrix metalloproteinase-2. ^*^p < 0.05 vs. controls.

We found significantly reduced plasma concentration of (total and active) MMP-2 in patients with asthma compared to a group of healthy volunteers (Figure 1). The level of the active form of MMP-2 in plasma as measured by zymography was also significantly decreased in patients with asthma compared to controls (Supplementary Figures 2A,B). The level of the active form of MMP-9 was not significantly different between asthma patients and controls (Supplementary Figures 2A,B). There was no significant difference in the plasma concentration of MMP-2 between patients grouped by disease severity (Supplementary Figure 3A). It is worth noting that inhaled corticosteroids may suppress the expression of MMP-2 (30). However, the plasma concentrations of MMP-2 were not significantly different between asthmatic patients receiving different doses of inhaled corticosteroids (Supplementary Figure 3B). The mean ages (62.2 ± 17.3 vs. 59.7 ± 7.0; *p* > 0.05) and the sex distribution ([M/F] 12/14 vs. 13/21; *p* > 0.7) between bronchial asthma patients and healthy subjects, respectively, were not significantly different. As expected, the plasma and BALF concentrations of MMP-2 significantly increased in hMMP-2 transgenic mice compared to their wild type counterparts (Supplementary Figure 4). The plasma and BALF concentrations of MMP-9 and the plasma concentration of TIMP-2 were significantly increased in hMMP-2 transgenic mice compared to their wild type counterparts (Supplementary Figure 4).

### Decreased Hyperresponsiveness in hMMP-2 TG Mice

**Figure 2:**
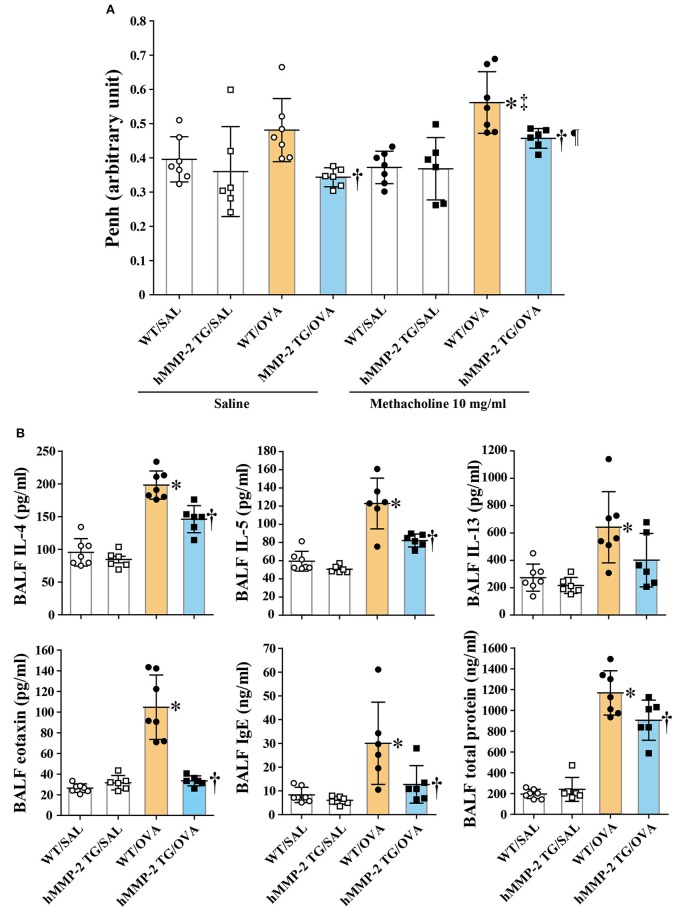
Decreased hyperresponsiveness and weak Th2 response in hMMP-2 TG mice. Wild type (WT) and proMMP-2 TG became allergic after sensitization and challenge with ovalbumin (OVA). Mice receiving saline were the controls. **(A)** Penh was measured using a plethysmography. **(B)** The concentrations of immunoglobulins, cytokines, and eotaxin were measured using commercial immunoassay kits. Bars indicate the means ± S.D. The figures are showing the combined results of two independent experiments. Statistical difference was evaluated by analysis of variance with Tukey test. Mann-Whitney *U*-test was used to compare Penh values within a group during saline and methacholine inhalation. hMMP-2, human matrix metalloproteinase-2; WT, wild type; SAL, saline; TG, transgenic; OVA, ovalbumin; IL, interleukin. WT/SAL with *n* = 7, hMMP-2 TG/SAL *n* = 6, WT/OVA *n* = 7, hMMP-2 TG/OVA *n* = 6. ^*^p < 0.05 vs. WT/SAL during methacholine inhalation; ^†^*p* < 0.05 vs. WT/OVA during inhalation of saline or methacholine; ^‡^*p* < 0.05 vs. WT/OVA during saline inhalation; ^¶^*p* < 0.05 vs. hMMP-2 TG/OVA during saline inhalation.

During saline inhalation, the Penh values were significantly different between WT/OVA and hMMP-2/OVA groups but not between other groups (Figure 2A). During inhalation of methacholine there was significant difference in Penh between WT/SAL and WT/OVA, and between WT/OVA and hMMP-2/OVA groups but not between other groups. Comparison of Penh values during saline and methacholine inhalation showed that both WT/OVA and hMMP-2/OVA groups have higher Penh values during methacholine inhalation than during saline inhalation (Figure 2A). However, due to limitations of the unrestrained plethysmography here these results should be interpreted with caution (31).

### Weak Increase of Cytokines in hMMP-2 TG Mice

The BALF concentrations of IL-4, IL-5, eotaxin, IgE, and total protein were significantly decreased, and the BALF level of IL-13 tended to be low in hMMP-2 TG/OVA mice compared to WT/OVA mice (Figure 2B). The lung tissue concentrations of IL-13 and IgE were significantly reduced while those of IL-4 and IL-5 tended to be low in hMMP-2 TG/OVA mice compared to WT/OVA mice (Supplementary Figure 5).

RT-PCR analysis of the lung tissue also revealed significantly reduced mRNA relative expression of IL-5, IL-13, and IL-10 in hMMP-2 TG/OVA mice compared to WT/OVA mice (Supplementary Figure 6).

The BALF and lung tissue concentration of MCP-3 and the plasma concentration of IgG2c were significantly decreased in hMMP-2 TG/OVA mice compared to WT/OVA mice (Supplementary Figure 7).

Evaluation of BALF cells revealed increased infiltration of leukocytes with a predominant count of eosinophils in WT/OVA and hMMP-2 TG/OVA mice compared to non-allergic control mice but they were significantly decreased in hMMP-2 TG/OVA compared to their WT counterparts (Supplementary Figures 8A,B). The plasma concentration of periostin, a matricellular protein commonly associated with eosinophilia significantly decreased in the hMMP-2 TG/OVA group compared to the WT/OVA group (Supplementary Figure 8C). There were no significant changes in the control saline groups.

### Reduced Infiltration of Inflammatory Cells and Mucin Stain in Lung Tissue From TG Mice

**Figure 3:**
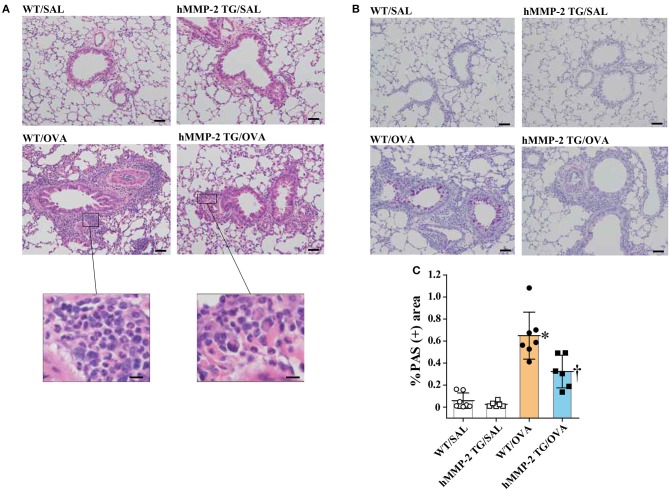
Reduced tissue eosinophilia and mucin secretion in hMMP-2 TG mice. Wild type (WT) and proMMP-2 TG became allergic after sensitization and challenge with ovalbumin (OVA). Mice receiving saline were the controls. Lung tissue was stained with hematoxylin & eosin **(A)** or with periodic acid-Schiff **(B)**. Quantification of periodic acid-Schiff positive area was performed using the WindROOF software **(C)**. Bars indicate the means ± S.D. Scale bars indicate 50 μm for the representative photomicrograph of each group, and 10 μm for the images further magnified in **(A)**. The figures are showing the combined results of two independent experiments. Statistical difference was evaluated by analysis of variance with Tukey test. hMMP-2, human matrix metalloproteinase-2; WT, wild type; SAL, saline; TG, transgenic; OVA, ovalbumin. WT/SAL with *n* = 7, hMMP-2 TG/SAL *n* = 6, WT/OVA *n* = 7, hMMP-2 TG/OVA *n* = 6. ^*^p < 0.05 vs. WT/SAL; ^†^*p* < 0.05 vs. WT/OVA.

We performed H&E staining and found significantly increased infiltration of leukocytes mainly comprised of eosinophils in intraalveolar spaces and peribronchial and peribronchiolar areas of the lungs from WT/OVA mice compared to hMMP-2 TG/OVA mice; infiltration of leukocytes was absent in saline control mice (Figure 3A). PAS staining showed hyperplasia of goblet cells in association with increased mucin secretion in the bronchial epithelium of WT/OVA compared to hMMP-2 TG/OVA mice; staining of the lung from control mice revealed normal findings (Figures 3B,C).

### Increased Akt Activation in the Lungs From TG Mice

There are reports showing that MMP-2 can directly inhibit cell apoptosis by activating the integrin/Akt axis (27). In line with this, we found significantly increased phosphorylation of Akt in lung from hMMP-2 TG/OVA group compared to the WT/OVA group (Supplementary Figure 9). There were no significant changes in the saline control groups.

### Systemic hMMP-2 Overexpression Exerts No Effect on T Cell Activation

To rule out whether the reduced allergic asthma is due to impaired adaptive immune response in the hMMP-2 TG mice, we isolated lymphocytes from spleen and evaluated T cell activation in response to allergen in WT and hMMP-2 TG mice sensitized and challenged with OVA or treated with saline. The proliferative activity of CD4^+^ and CD8^+^ T cells in the presence of OVA was similar in both WT and hMMP-2 TG mice (Supplementary Figures 10A,B). In addition, T cells from the spleen of hMMP-2 TG mice were significantly activated in the presence of the OVA compared to cells cultured in the presence of saline (Supplementary Table 3). Interestingly, there was a robust and significant Th1 response in T cells from hMMP-2 TG/OVA mice compared to T cells from WT/OVA mice (Supplementary Table 3).

### An Inhibitor of Matrix Metalloproteinases Abolished the Beneficial Effect of hMMP-2 Overexpression

**Figure 4:**
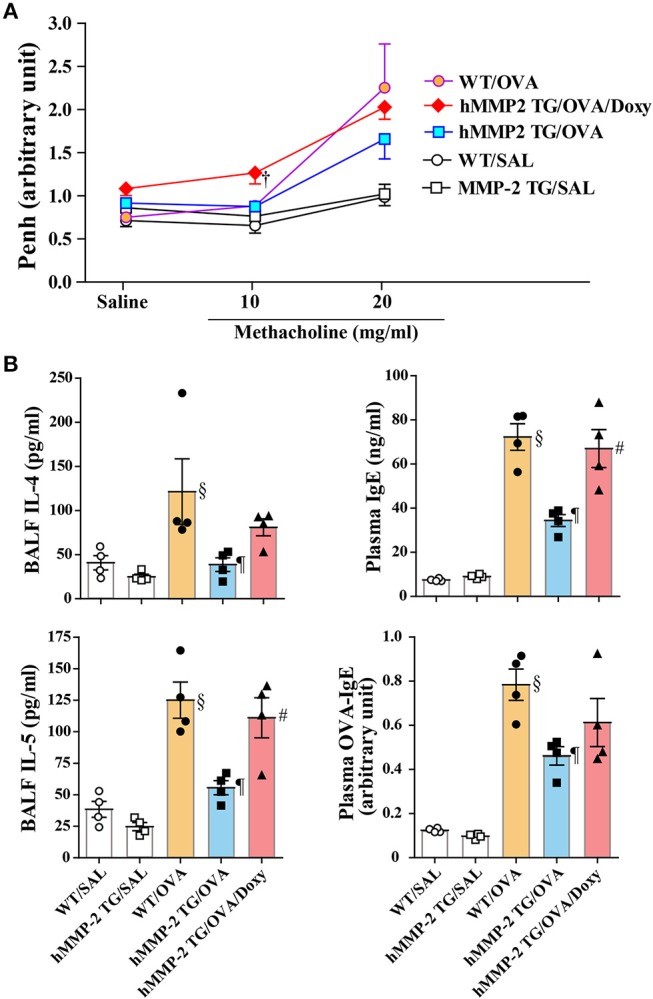
An inhibitor of matrix metalloproteinases blocks the beneficial effect of hMMP-2 overexpression. Wild type (WT) and proMMP-2 TG became allergic after sensitization and challenge with ovalbumin (OVA). A group of hMMP-2 TG mice was fed with baits containing 5% doxycycline before, during sensitization and challenge with OVA. Mice receiving saline were the controls. **(A)** Penh was measured using a plethysmography. **(B)** The concentrations of immunoglobulins and cytokines were measured using commercial immunoassay kits. Bars indicate the means ± S.E.M. Statistical difference was evaluated by analysis of variance with Tukey test. Mann-Whitney *U*-test was used to compare Penh values within a group during saline and methacholine inhalation. hMMP-2, human matrix metalloproteinase-2; WT, wild type; SAL, saline; TG, transgenic; OVA, ovalbumin; IL, interleukin; Doxy, doxycycline. WT/SAL with *n* = 4, hMMP-2 TG/SAL *n* = 4, WT/OVA *n* = 4, hMMP-2 TG/OVA *n* = 4, hMMP-2 TG/OVA/Doxy *n* = 4. ^†^*p* < 0.05 vs. counterpart hMMP-2/OVA during methacholine inhalation; ^§^*p* < 0.05 vs. WT/SAL; ^¶^*p* < 0.05 vs. WT/OVA; ^#^*p* < 0.05 vs. hMMP-2 TG/OVA.

A group of hMMP-2 TG mice was fed with doxycycline, an inhibitor of matrix metalloproteinases, for 2 weeks before sensitization and challenge with OVA and the allergic response was compared with other groups of mice fed with standard food. After saline inhalation, the Penh values were not significantly different between groups. The airway responsiveness was significantly increased in the hMMP-2 TG/OVA/Doxy group compared to the hMMP-2 TG/OVA group after inhalation of 10 mg/ml methacholine. The hMMP-2 TG/OVA group showed reduced airway responsiveness compared to both WT/OVA and hMMP-2 TG/OVA/Doxy groups after inhalation of 20 mg/ml methacholine although the reduction was not statistically significant (Figure 4A).

The BALF levels of IL-4 and IL-5, and the plasma levels of total IgE and OVA-specific IgE were significantly decreased in hMMP-2/OVA compared to WT/OVA (Figure 4B). The BALF levels of IL-5 and plasma level of total IgE were significantly elevated and the BALF levels of IL-4 and plasma level of OVA-specific IgE tended to be high in the hMMP-2 TG/OVA/Doxy group compared to the hMMP-2 TG/OVA group (Figure 4B). The BALF levels of total IgE, OVA-specific IgE, and IFNγ were also different between hMMP-2 TG/OVA/Doxy and hMMP-2 TG/OVA groups but they did not reach significant difference (Supplementary Figures 11A,B).

### Polarization of Macrophages Toward M1 Phenotype by Human MMP-2

**Figure 5:**
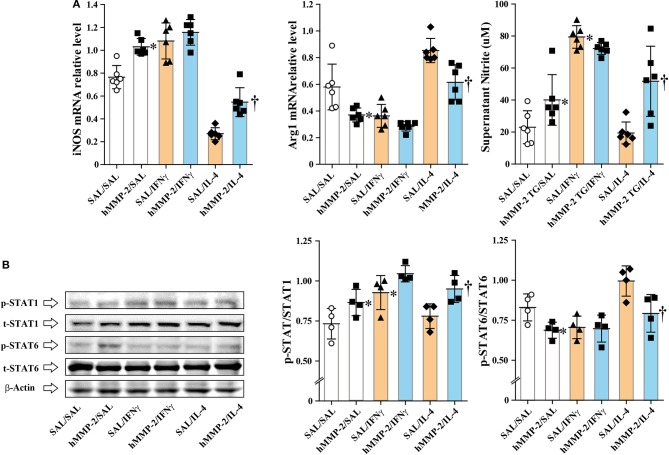
Human MMP-2 promotes polarization of macrophages toward M1 phenotype. The mouse macrophage cell line IC-21 was cultured in the presence or absence of active human MMP-2, and then IL-4 and IFNγ were added to the culture media. **(A)** The relative mRNA expression of inducible nitric oxide synthase (iNOS) and arginase 1 (Arg1) was evaluated by reverse-transcriptase polymerase chain reaction. **(B)** Phosphorylation of STAT1 and STAT6 was assessed by Western blotting. Two independent experiments were performed and the results of one experiment are shown. *N* = 4 in each group. Bars indicate the means ± S.D. Statistical difference was evaluated by analysis of variance with Tukey test. hMMP-2, human matrix metalloproteinase-2. ^*^p < 0.05 vs. SAL/SAL; ^†^*p* < 0.05 vs. SAL/IL-4.

We cultured the mouse macrophage cell line IC-21 in the presence of active human MMP-2, and then treated with IFNγ and IL-4 (M2 polarizing condition). The addition of MMP-2 to cell culture significantly increased the relative mRNA expression of inducible nitric oxide synthase (iNOS) but decreased that of arginase 1 (Arg1) compared to untreated cells (Figure 5A). The concentration of nitrite ion significantly increased in supernatants from cells cultured treated with MMP-2 compared to untreated cells. In addition, MMP-2 treatment was associated with significant enhancement of STAT1 phosphorylation and decreased STAT6 phosphorylation compared to untreated conditions (Figure 5B).

**Figure 6:**
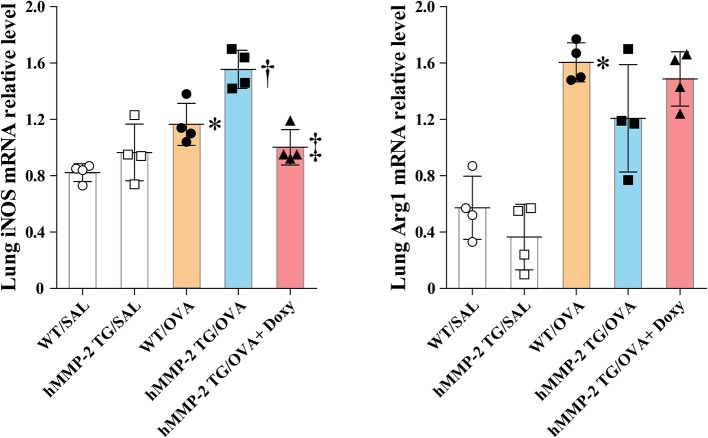
Increased markers of M1 polarization in lung tissue from hMMP-2 transgenic mice. Wild type (WT) and proMMP-2 TG became allergic after sensitization and challenge with ovalbumin (OVA). A group of hMMP-2 TG mice was fed with baits containing 5% doxycycline before, during sensitization, and challenge with OVA. Mice receiving saline were the controls. The relative mRNA expression of inducible nitric oxide synthase (iNOS) and arginase 1 (Arg1) was evaluated by reverse-transcriptase polymerase chain reaction. The figures are showing the results of one independent experiment. Bars indicate the means ± S.D. Statistical difference was evaluated by analysis of variance with Tukey test. hMMP-2, human matrix metalloproteinase-2. WT/SAL with *n* = 4, hMMP-2 TG/SAL *n* = 4, WT/OVA *n* = 4, hMMP-2 TG/OVA *n* = 4, hMMP-2 TG/OVA/Doxy *n* = 4. ^*^p < 0.05 vs. WT/SAL; ^†^*p* < 0.05 vs. WT/OVA; ^‡^*p* < 0.05 vs. hMMP-2 TG/OVA.

The mRNA relative expression of iNOS and Arg1 was also evaluated in lung tissue and BALF cells from WT and hMMP-2 TG mice sensitized and challenged with OVA. In lung tissue, the mRNA expression of iNOS was significantly increased in hMMP-2 TG/OVA mice compared to WT/OVA mice but it was significantly decreased in hMMP-2 TG/OVA/Doxy mice (Figure 6). The mRNA expression of Arg-1 in lung tissue tended to be low in hMMP-2 TG/OVA compared to WT/OVA mice (Figure 6). In BALF cells, the mRNA expression of iNOS tended to be high in hMMP-2 TG/OVA compared to WT/OVA mice, whereas the Arg1 mRNA expression was significantly decreased in hMMP-2 TG/OVA compared to WT/OVA mice (Supplementary Figure 12). In addition, phosphorylation of STAT1 in lung tissue was significantly increased whereas that STAT6 was significantly decreased in hMMP-2 TG/OVA mice compared to WT/OVA (Supplementary Figure 13).

**Figure 7:**
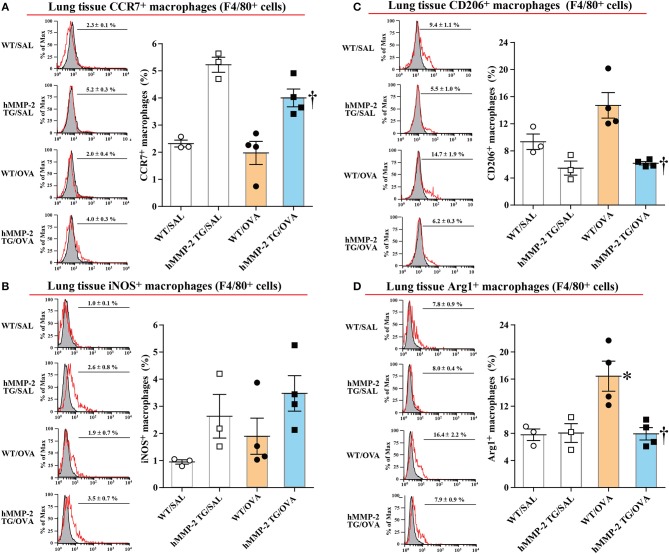
Increased percentage of M1 macrophages in lung tissue from hMMP-2 transgenic mice. Wild type (WT) and proMMP-2 TG became allergic after sensitization and challenge with ovalbumin (OVA). Mice receiving saline were the controls. The percentage of CCR7^+^ **(A)**, iNOS **(B)**, CD206^+^ **(C)**, and Arg1 **(D)** was evaluated by flow cytometry. The figures are showing the results of one independent experiment. Bars indicate the means ± S.E.M. Statistical difference was evaluated by analysis of variance with Tukey test. hMMP-2, human matrix metalloproteinase-2. WT/SAL with *n* = 3, hMMP-2 TG/SAL *n* = 3, WT/OVA *n* = 4, hMMP-2 TG/OVA *n* = 4. ^*^p < 0.05 vs. WT/SAL; ^†^*p* < 0.05 vs. WT/OVA.

The M1 polarization of lung tissue and BALF macrophages in allergen-sensitized and -challenged mice was also evaluated *in vivo*. In this separate experiment, hMMP-2 TG/OVA mice also showed significant decrease of airway responsiveness compared to their WT counterparts (Supplementary Figure 14). We then evaluated by flow cytometry the percentage of cells with surface expression of CCR7, a M1 marker, and CD206, a M2 marker, and with intracellular levels of iNOS and Arg1 in F4/80^+^ gated lung cells. The percentage of CCR7^+^ and iNOS^+^ macrophages was increased while the percentage of CD206^+^ and Arg1^+^ macrophages were significantly decreased in lung tissue and BALF from hMMP-2 TG/OVA mice compared to WT/OVA mice (Figures 7A–D; Supplementary Figures 15A–D).

### No Change in the Percentage of Regulatory T Cells in Allergic Mice Overexpressing hMMP-2

Regulatory T cells are characterized by the surface expression of CD4 and CD25. Here we compared the percentage of CD4^+^CD25^+^ T cells between hMMP-2 TG/OVA and WT/OVA and the results showed no significant different between groups (Supplementary Figures 16A–D).

## Discussion

### Lung Inflammation in MMP-2-Deficient, -Sufficient, and -Overexpressing Mice

A growing amount of evidence points to airway inflammation playing a key role in the pathogenesis of asthma that results from the interplay between genetic and environmental components (32). The clinical syndrome of asthma includes breathlessness, hyperresponsiveness, wheezing, and cough (3, 32). There are several types of asthma but the most common form is allergy-associated (33). There is airway infiltration of a selective subpopulation of activated helper-T lymphocytes, Th2 cells, which secrete high quantity of cytokines such as IL-4, IL-5, and IL-13 that, in turn, stimulate the activation and recruitment of eosinophils, mast cells and B cells, which produce and release allergen-specific IgE (32, 34). In line with previous observations, in the present study, allergic wild type mice showed increased lung infiltration of leukocytes, in particular of eosinophils, hyperplasia of lung mucin-secreting cells, elevated lung concentration of Th2 cytokines and IgE with significant airway hyperresponsiveness compared to control mice. Interestingly, allergen-sensitized and -challenged transgenic mice overexpressing human MMP-2 had significantly less eosinophils, mucin-secreting cells and concentration of Th2 cytokines, eotaxin, and IgE in the lungs together with significantly reduced airway hyperresponsiveness compared to their wild type counterparts, suggesting that human MMP-2 is protective against allergic bronchial asthma. The significant increased number of inflammatory cells with high expression level of Th2 cytokines in the lungs, and the significant high mortality rate observed in MMP-2-deficient mice with allergic lung inflammation compared to allergic wild type mice supports the assumption that MMP-2 protects against allergic asthma (35). Blockade of the beneficial effect of hMMP-2 overexpression by an inhibitor of matrix metalloproteinase observed in our present study further supports the protective activity of hMMP-2 in allergic asthma. In this context and considering the low plasma level of MMP-2 in our asthmatic patients compared to controls, it may be permissible to speculate that low MMP-2 is associated with susceptibility to allergic asthma.

### Tilting of M1/M2 Balance Toward M1 Phenotype

Several studies have shown that a group of matrix metalloproteinases affects M1/M2 polarization of macrophages (36). M1 macrophages play a critical function against microbial infection by releasing inflammatory cytokines (e.g., IL-12), reactive oxygen species, and reactive nitrogen species (37, 38). By contrast, M2 macrophages secrete anti-inflammatory cytokines (e.g., IL-10), growth factors, matrix proteins, and arginase 1 (Arg1) which competes with inducible nitric synthase (iNOS) for the substrate arginine (37, 38). The balance between iNOS/Arg1 is tilted in favor of iNOS in M1, and in favor of Arg1 in M2 macrophages (39). Polarization of macrophages toward the M1 phenotype downregulates Th2 cytokine expression (37). These prior observations led us to hypothesize that MMP-2 protects against allergic asthma because it downregulates Th2 immune response by promoting the differentiation of macrophages to M1 phenotype. To demonstrate this hypothesis we cultured a macrophage cell line *in vitro* and assessed whether active human MMP-2 can reverse markers of M1 (iNOS) and M2 (Arg1) macrophages under IL-4 stimulation. MMP-2 significantly increased the expression of iNOS but reduced that of Arg1 in macrophages. In line with this, Western blot analysis showed significant activation of STAT1, a M1 stimulator, and decreased activation of STAT6, a M2 stimulator, in macrophages treated with human MMP-2 (40). In agreement with these *in vitro* observations, there was significantly increased percentage of CCR7^+^ macrophages with increased iNOS expression and significantly reduced percentage of CD206^+^ macrophages with decreased Arg1 expression in the lungs from allergic mice overexpressing hMMP-2 compared with their WT counterparts. Overall, these results suggest that stimulation of M1 differentiation by MMP-2 is one possible explanation for the protective activity of human MMP-2 overexpression in allergic asthma.

### Alternative Mechanisms

There is experimental evidence showing that lack of MMP-2 is associated with excessive cell apoptosis in the lungs with allergic inflammation and, in this connection, a recent report demonstrated that hMMP-2 directly inhibits apoptosis of varying cells by binding to cell surface integrin-3 leading to activation of the anti-apoptotic Akt pathway (20, 27). Another report has shown that MMP-2 cleaves and produces fragments of Th2 chemokines including chemokine (C-C motif) ligand 7 (CCL7) or monocyte-chemotactic protein-3 (MCP-3), CC-motif chemokine 11 (CCL11) or eotaxin-1, and chemokine (C-C motif) ligand 17 (CCL17) or thymus and activation regulated chemokine (TARC), that play important roles in the induction of allergic inflammation in asthmatic conditions (41, 42). In support of these findings, here we found a significant low level of the Th2 chemokine MCP-3 and increased activation of the Akt pathway in allergic hMMP-2 transgenic mice compared to their wild type counterparts. Therefore, suppression of apoptosis of M1 macrophages or Th1 cells, and alteration of biochemical properties of Th2 chemokines by proteolytic processing may be other alternative mechanisms mediating the protective activity of hMMP-2 in allergic asthma. Mediation of regulatory T cells is another alternative mechanism but there was no significant difference in the percentage of CD4^+^CD25^+^ cells between allergen-sensitized and challenged hMMP-2 transgenic mice and its counterpart WT mice.

### Limitations and Strengths

The scarce number of patients, the retrospective nature of the clinical study, the lack of experiments demonstrating that exogenous MMP-2 is also beneficial for lung inflammation and measurement of airway hyperresponsiveness using only enhanced pause are limitations of the current study. However, it is important to note that a previous study showing that MMP-2 knockout aggravates lung inflammation supports the results of the present study (35).

## Conclusion

In brief, the present investigation showed that hMMP-2 protects the lung against allergic inflammation by promoting differentiation of M1 macrophages. This finding reveals a novel and potential approach for the management of allergic inflammation in asthma.

## Ethics Statement

The institutional review board of Mie University approved the clinical study protocol (Approval No. 2846; February 19, 2015). The Mie University's Committee on Animal Investigation approved the experimental protocol (Approval No. 25-20/Hen1-Sai; September 12, 2015).

## Author Contributions

YTakahashi, CD'A-G, and KF performed and prepared the allergic asthma mouse models. HF and KA performed and analyzed the lung function test. MT, TY, KN, RI, AT, and TO measured and analyzed several parameters. LQ and YTakei contributed with critical revision and interpretation of the data. TK, EG, and OT conceived and designed the experiments, analyzed the data and contributed with critical revision and interpretation.

### Conflict of Interest Statement

The authors declare that the research was conducted in the absence of any commercial or financial relationships that could be construed as a potential conflict of interest.
