# Supplementary material for: Protective Role of Matrix Metalloproteinase-2 in Allergic Bronchial Asthma
Source: Front Immunol. 2019 Aug 2;10:1795. doi: 10.3389/fimmu.2019.01795 (PMC6687911; doi:10.3389/fimmu.2019.01795)
Supplement: Supplementary file 1 [file Data_Sheet_1.PDF]

Supplementary Figures 1 to 16 and Supplementary Tables 1 to 3

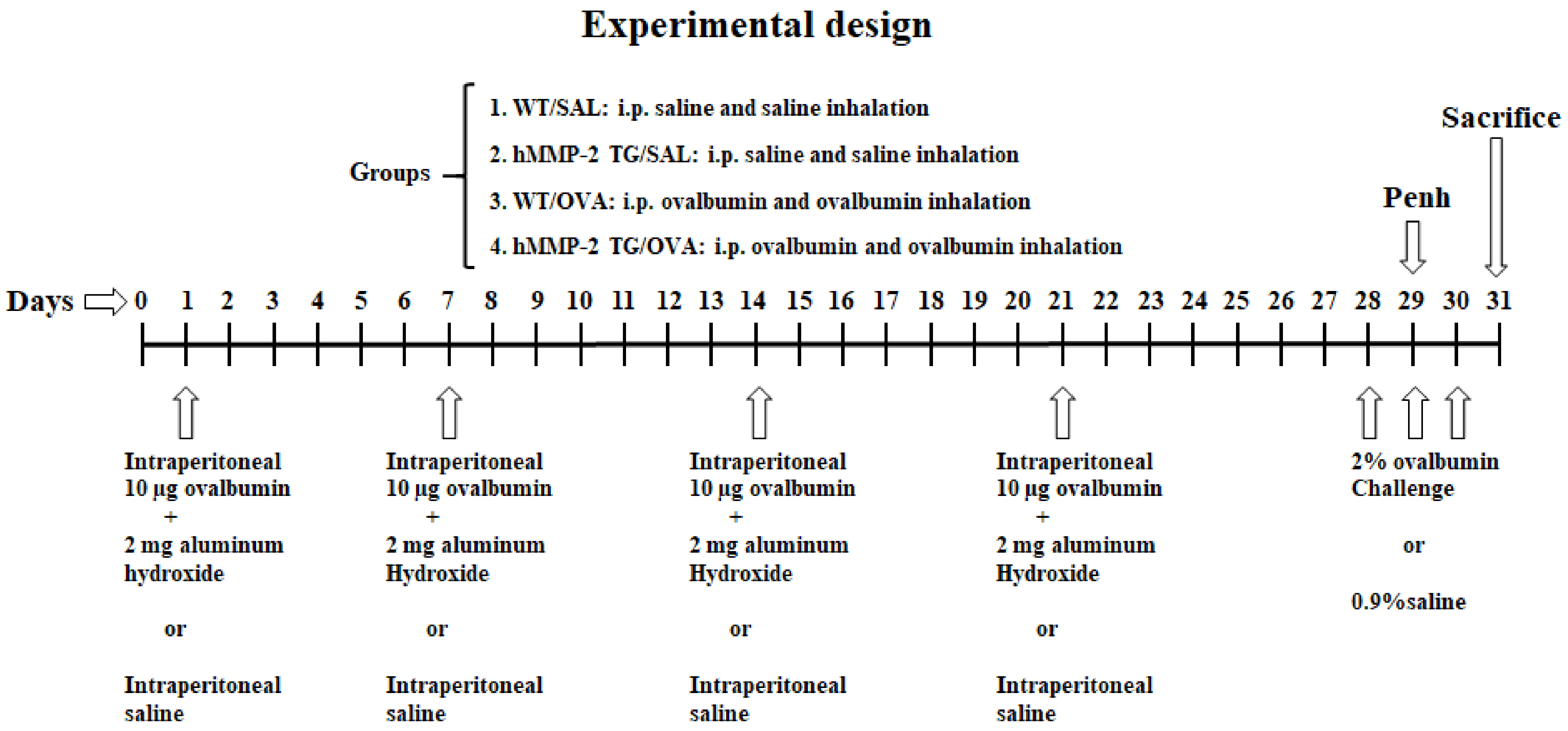

Supplementary Fig. 1. Experimental design of the asthma mouse model. The mouse model allergic bronchial asthma was developed as described under material and methods. Mice were sensitized four times with 10 µg of ovalbumin and 2 mg aluminum hydroxide, and then challenged three times with aerosolized 2% ovalbumin before sacrifice. Mice were categorized in four experimental groups. WT, wild type; SAL, saline; hMMP-2 TG, human matrix metalloproteiase-2 transgenic; i.p., intraperitoneal.

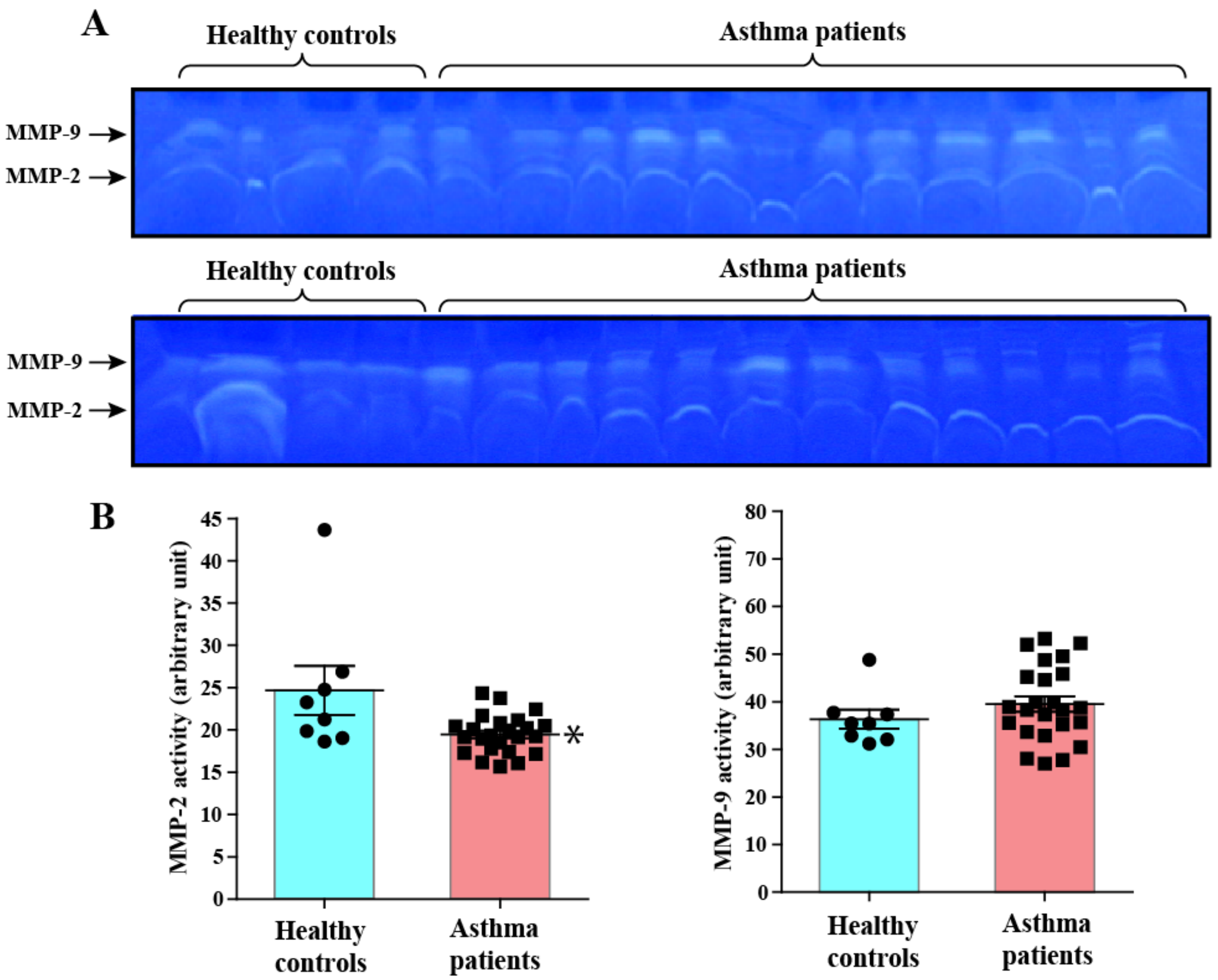

Supplementary Fig. 2. Significant reduction of the plasma level of active matrix metalloproteinase-2 in patients with asthma compared to healthy controls. The activity of matrix metalloproteinase (MMP)-2 and MMP-9 in plasma of 7 healthy subjects and in 24 patients with bronchial asthma was measured by zymography. Bars indicate the means ± S.E.M. Statistical difference was evaluated by Mann-Whitney U test. MMP-2, human matrix metalloproteinase-2. *p<0.05 vs controls.

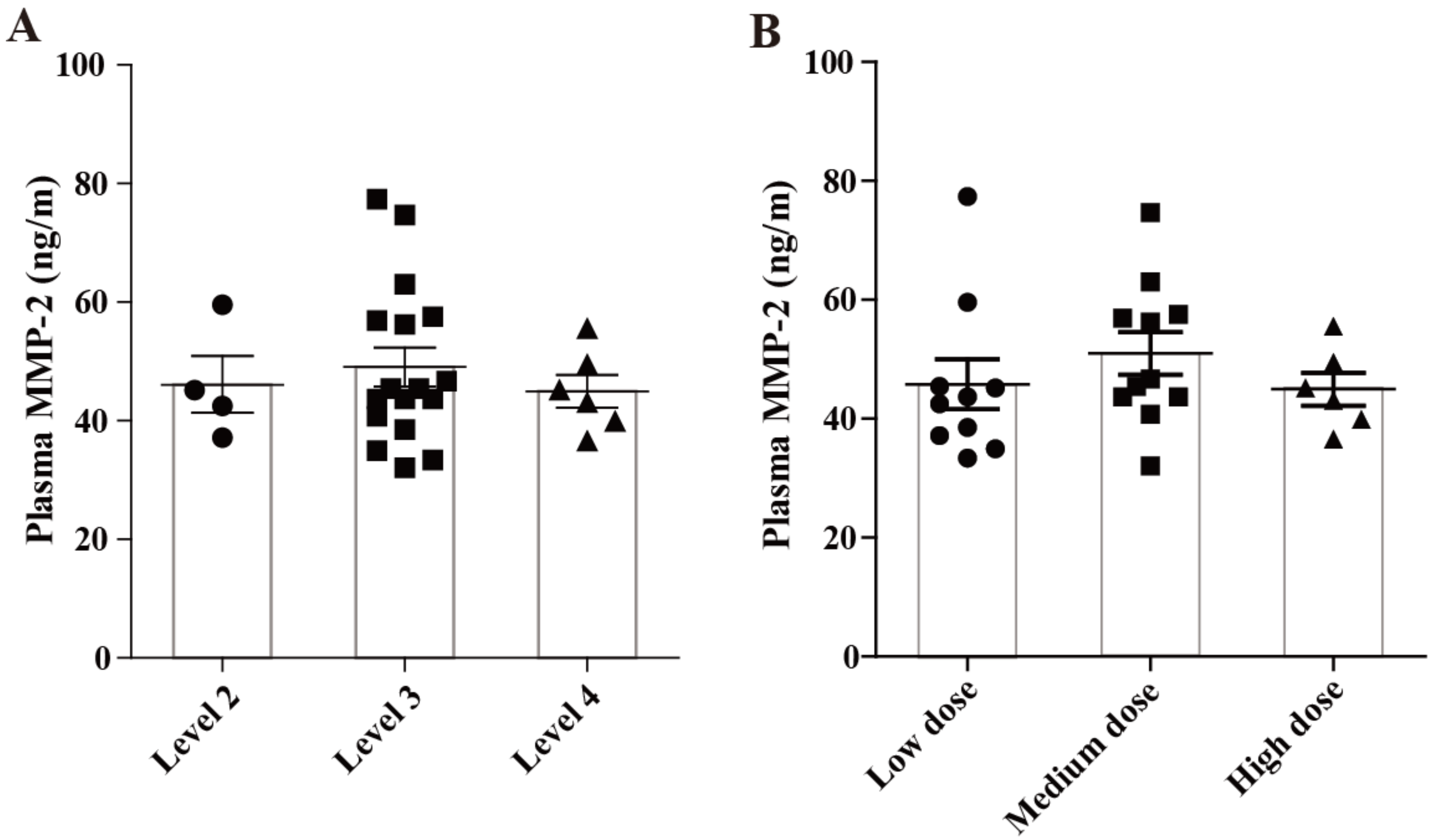

Supplementary Fig. 3. Dose of inhaled corticosteroids and disease severity exert no effect on plasma levels of MMP-2. Patients with bronchial asthma were grouped by the dose of inhaled corticosteroids (**A**) and disease severity (**B**) based on the GINA classification and the plasma levels of MMP-2 were compared. The levels of MMP-2 were measured using commercial immunoassay kits. Bars indicate the means ± S.E.M. Statistical difference was evaluated by analysis of variance with Tukey test. MMP-2, human matrix metalloproteinase-2. *p<0.05 vs controls.

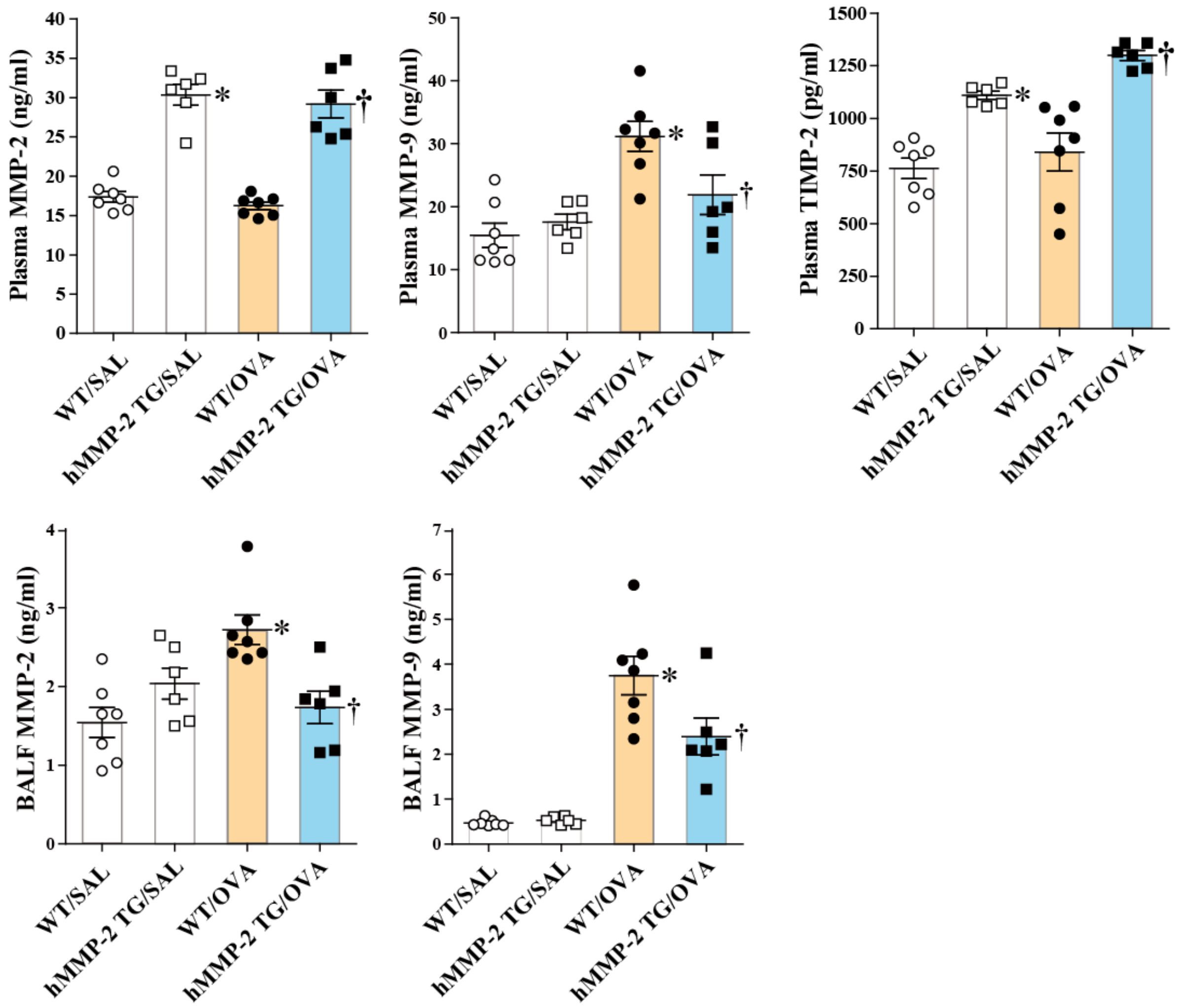

Supplementary Fig. 4. Increased concentrations of MMP-2, MMP-9 and TIMP-2 in hMMP-2 transgenic mice with allergic bronchial asthma. Wild type (WT) and proMMP-2 TG became allergic after sensitization and challenge with ovalbumin (OVA). Mice receiving saline were the controls. The levels of MMP-2, MMP-9 and TIMP-2 were measured using commercial immunoassay kits. Bars indicate the means ± S.E.M. The figures are showing the combined results of two separate experiments. Statistical difference was evaluated by analysis of variance with Tukey test. hMMP-2, human matrix metalloproteinase-2; TIMP-2, tissue inhibitor of metalloproteinase-2; MMP-9, matrix metalloproteinase-9; WT, wild type; SAL, saline; TG, transgenic; OVA, ovalbumin. WT/SAL with n=7, hMMP-2 TG/SAL n=6, WT/OVA n=7, hMMP-2 TG/OVA n=6. *$p<0.05$ vs WT/SAL; †$p<0.05$ vs WT/OVA.

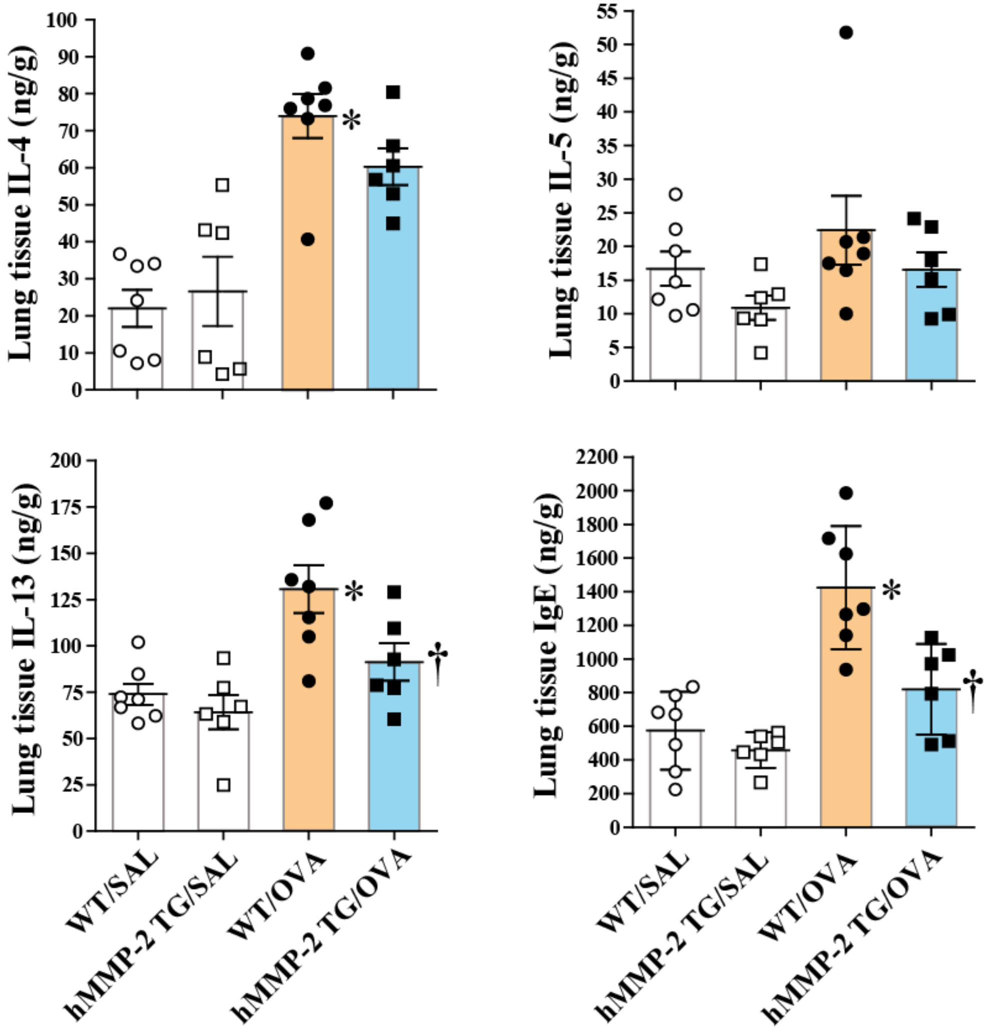

Supplementary Fig. 5. Reduced Th2 response in hMMP-2 TG mice. Wild type (WT) and proMMP-2 TG became allergic after sensitization and challenge with ovalbumin (OVA). Mice receiving saline were the controls. The concentrations of Th2 cytokines and immunoglobulins were measured using commercial immunoassay kits. Bars indicate the means ± S.E.M. The figures are showing the combined results of two separate experiments. Statistical difference was evaluated by analysis of variance with Tukey test. hMMP-2, human matrix metalloproteinase-2; WT, wild type; SAL, saline; TG, transgenic; OVA, ovalbumin; IL, interleukin. WT/SAL with n=7, hMMP-2 TG/SAL n=6, WT/OVA n=7, hMMP-2 TG/OVA n=6. *p<0.05 vs WT/SAL; †p<0.05 vs WT/OVA.

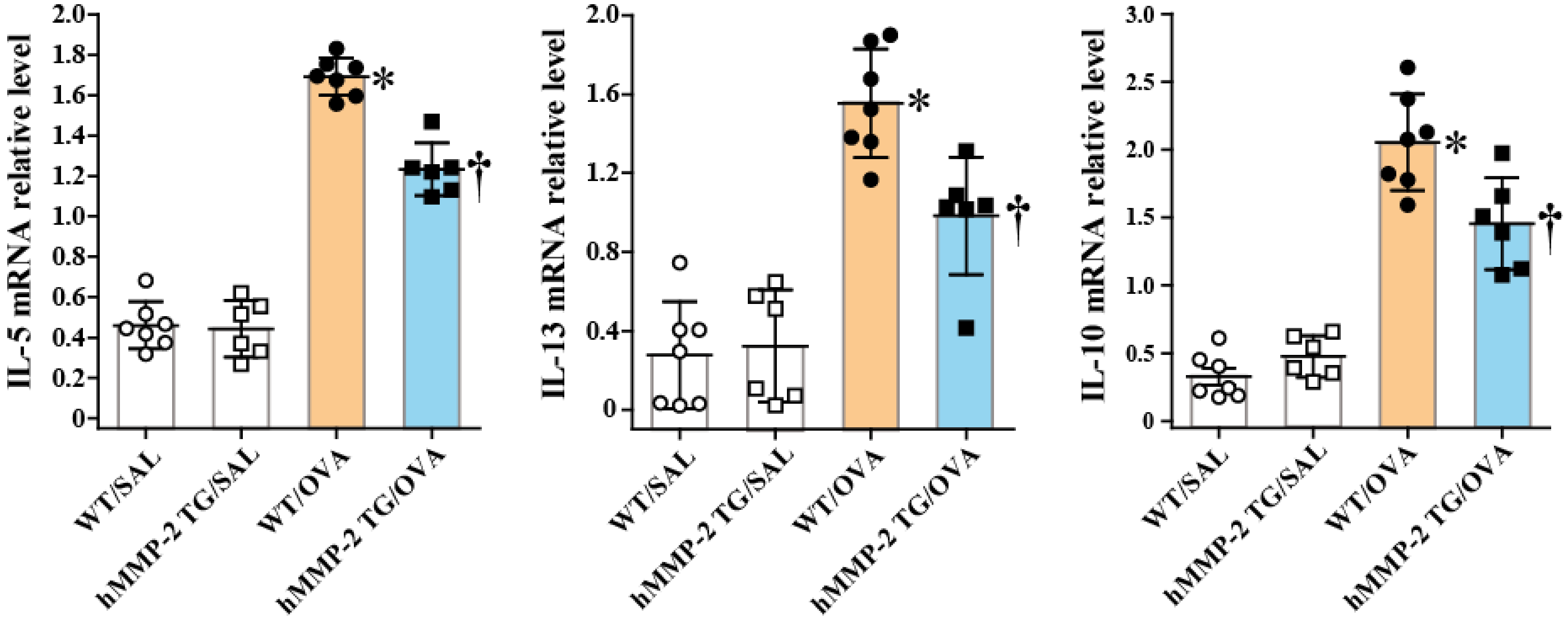

Supplementary Fig. 6. Decreased relative mRNA expression of IL-5, IL-13 and IL-10 in hMMP-2 TG mice. Wild type (WT) and proMMP-2 TG became allergic after sensitization and challenge with ovalbumin (OVA). Mice receiving saline were the controls. The mRNA expression of each cytokines was evaluated by reverse-transcriptase polymerase chain reaction. Bars indicate the means ± S.D. The figures are showing the combined results of two separate experiments. Statistical difference was evaluated by analysis of variance with Tukey test.hMMP-2, human matrix metalloproteinase-2; WT, wild type; SAL, saline; TG, transgenic; OVA, ovalbumin; IL, interleukin. WT/SAL with n=7, hMMP-2 TG/SAL n=6, WT/OVA n=7, hMMP-2 TG/OVA n=6. *$p<0.05$ vs WT/SAL; †$p<0.05$ vs WT/OVA.

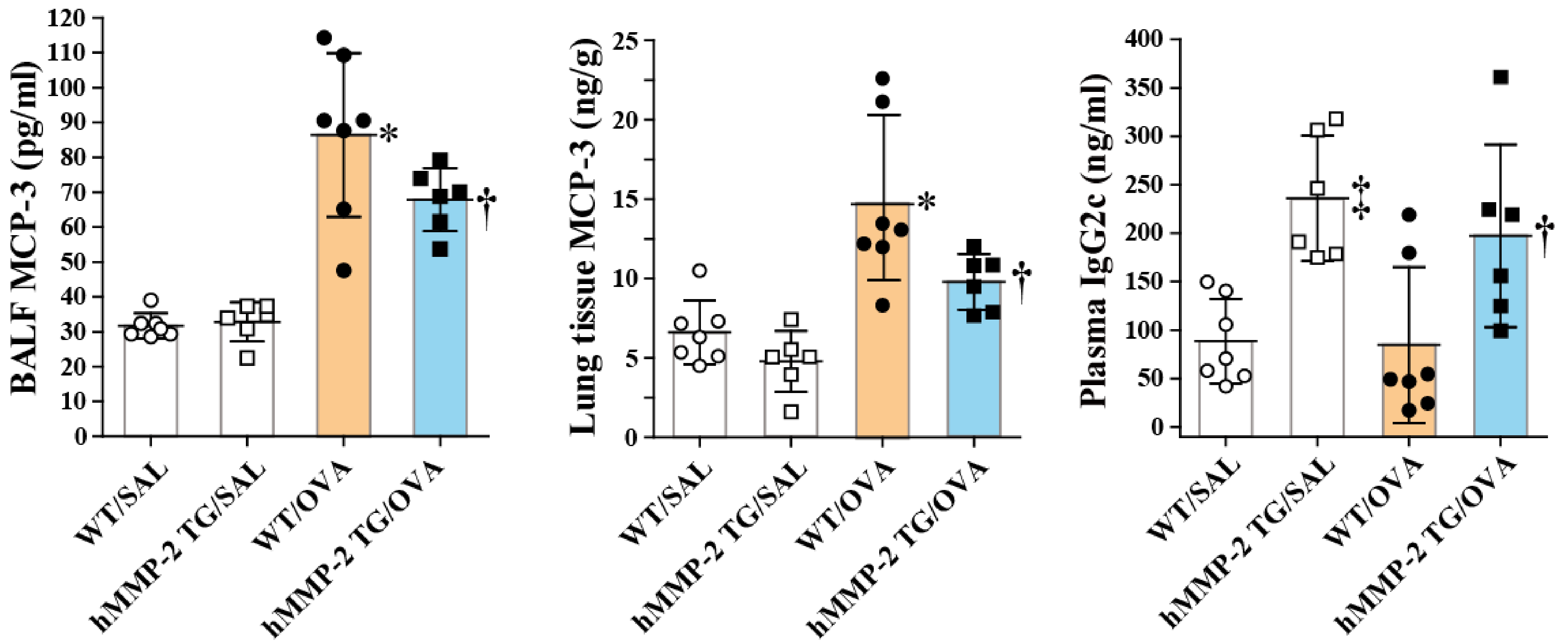

Supplementary Fig. 7. Decreased level of MCP-3 and increased level of IgG2c in hMMP-2 TG mice. Wild type (WT) and proMMP-2 TG became allergic after sensitization and challenge with ovalbumin (OVA). Mice receiving saline were the controls. The concentrations of MCP-3 and IgG2c were measured using commercial immunoassay kits. Bars indicate the means ± S.D. The figures are showing the combined results of two separate experiments. Statistical difference was evaluated by analysis of variance with Tukey test. hMMP-2, human matrix metalloproteinase-2; WT, wild type; SAL, saline; TG, transgenic; OVA, ovalbumin; MCP-3, monocyte chemoattractant protein-3. WT/SAL with n=7, hMMP-2 TG/SAL n=6, WT/OVA n=7, hMMP-2 TG/OVA n=6. *p<0.05 vs WT/SAL; †p<0.05 vs WT/OVA.

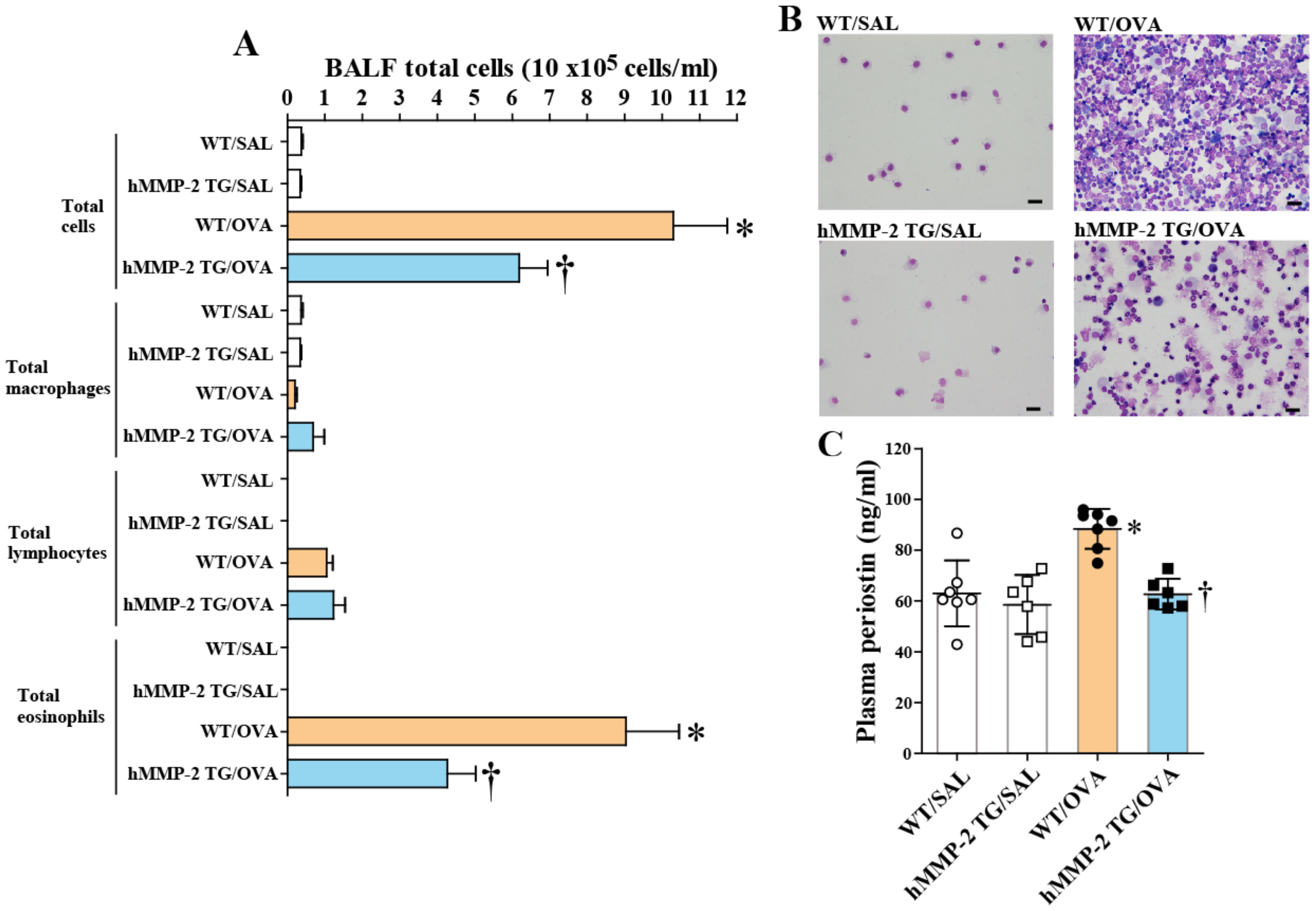

Supplementary Fig. 8. Decreased eosinophilic inflammation and plasma periostin level in hMMP-2 TG mice. Wild type (WT) and proMMP-2 TG became allergic after sensitization and challenge with ovalbumin (OVA). Mice receiving saline were the controls. Bronchoalveolar lavage fluid (BALF) cells were counted (A) and stained with May-Grünwald Giemsa for differential counting (B). Periostin was measured by enzyme immunoassays (C). Bars indicate the means ± S.D. Scale bars indicate 20 µm. The figures are showing the combined results of two separate experiments. Statistical difference was evaluated by analysis of variance with Tukey test. hMMP-2,hMMP-2, human matrix metalloproteinase-2; WT, wild type; SAL, saline; TG, transgenic; OVA, ovalbumin. WT/SAL with n=7, hMMP-2 TG/SAL n=6, WT/OVA n=7, hMMP-2 TG/OVA n=6. *p<0.05 vs WT/SAL; †p<0.05 vs WT/OVA

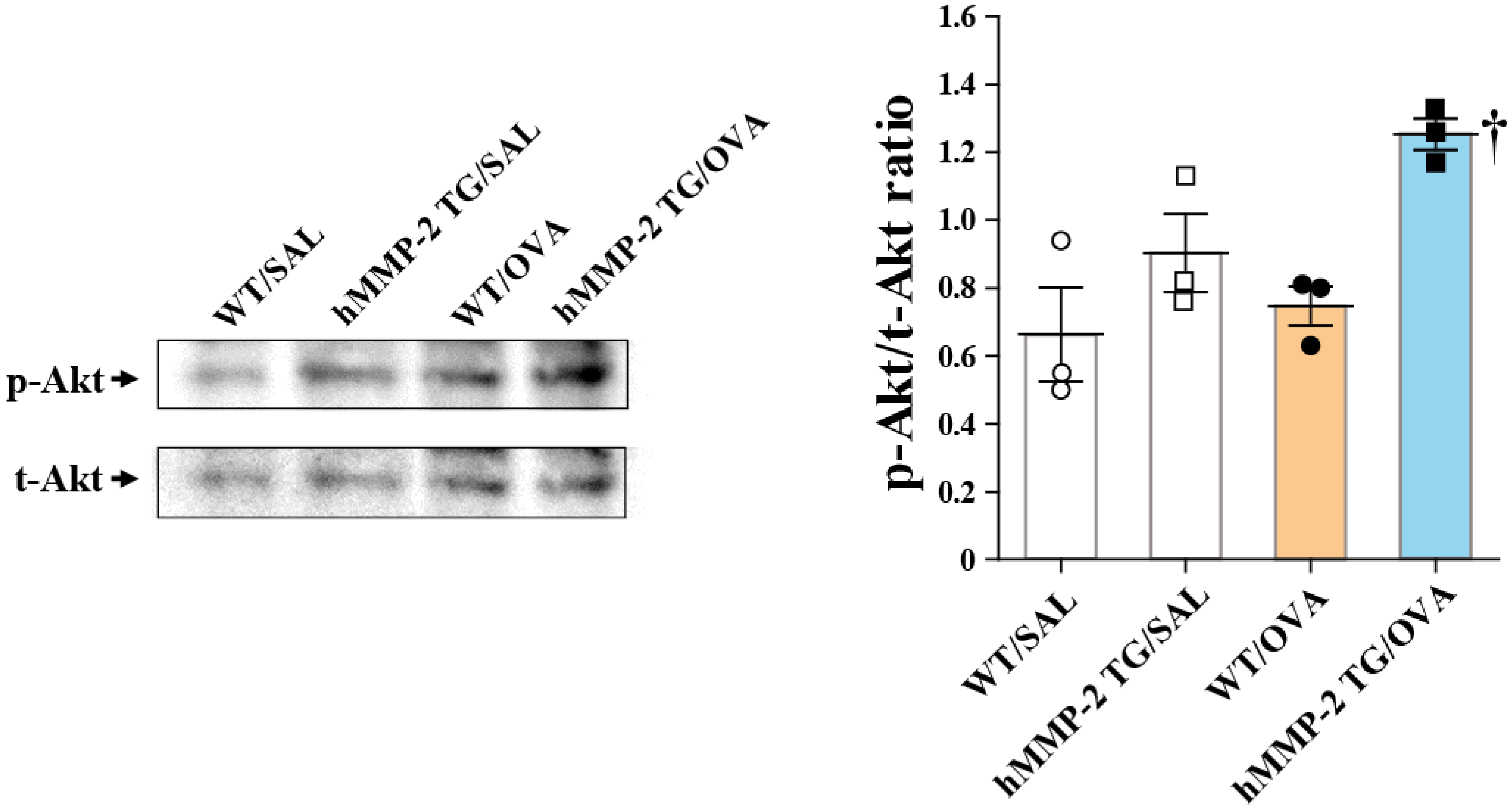

Supplementary Fig. 9. Increased Akt activation in hMMP-2 TG mice. Wild type (WT) and proMMP-2 TG became allergic after sensitization and challenge with ovalbumin (OVA). Mice receiving saline were the controls. Similar amount of protein was loaded on gel from lung tissue samples of each group and Western blotting was carried out. Bars indicate the means ± S.D. The figures are showing the combined results of two separate experiments. Statistical difference was evaluated by analysis of variance with Turkey test.hMMP-2, human matrix metalloproteinase-2; WT, wild type; SAL, saline; TG, transgenic; OVA, ovalbumin; p-Akt, phosphorylated Akt; t-Akt, total Akt. WT/SAL with n=3, hMMP-2 TG/SAL n=3, WT/OVA n=3, hMMP-2 TG/OVA n=3. *$p<0.05$ vs WT/SAL; †$p<0.05$ vs WT/OVA.

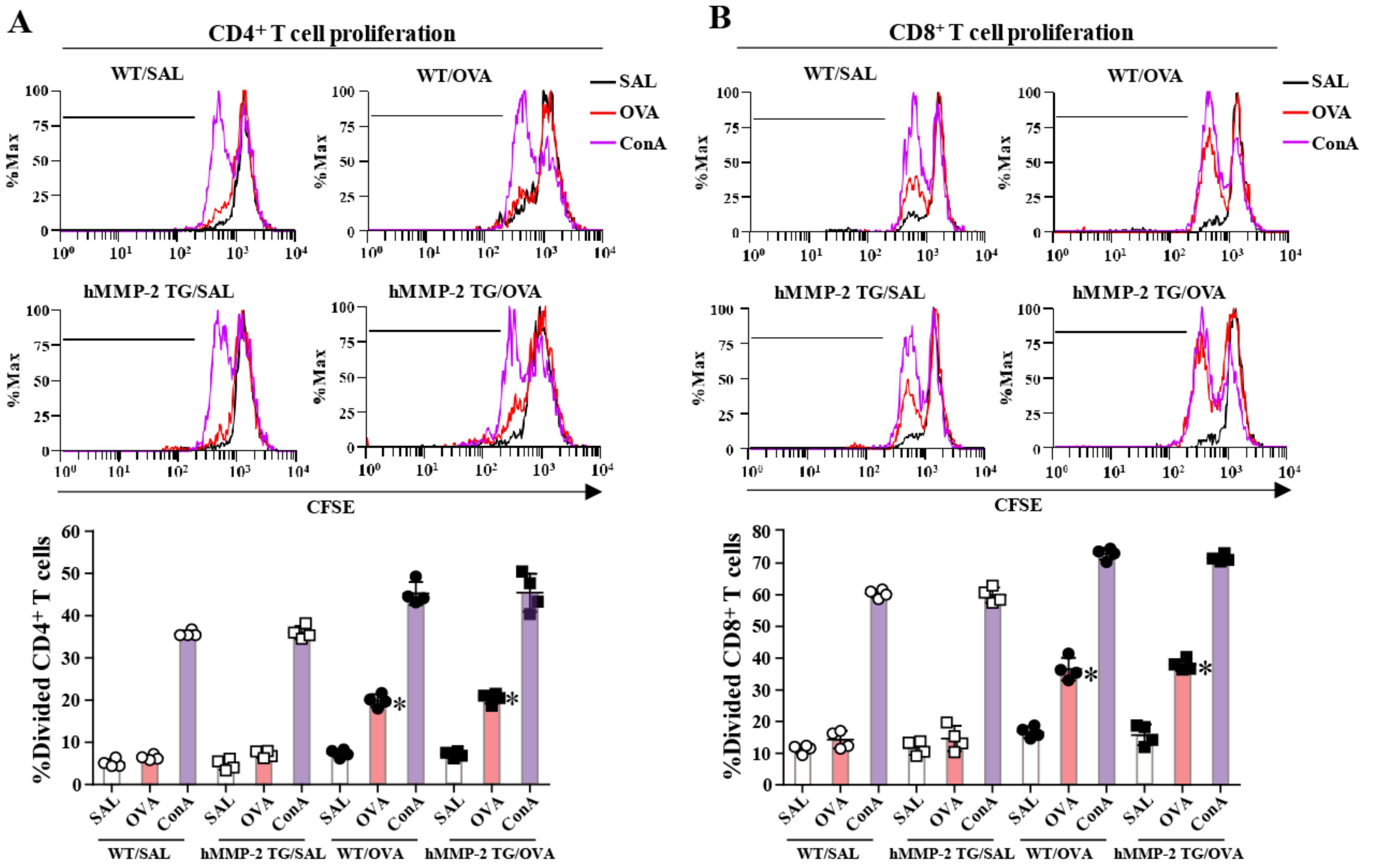

Supplementary Fig. 10. Human matrix metalloproteinase-2 (hMMP-2) overexpression exerts no effect on T cell proliferation. Spleen was excised from wild type (WT) and hMMP-2 transgenic (TG) mice sensitized and challenged with ovalbumin (OVA). Spleen cells were collected and analyzed ex vivo for OVA-specific T-cell proliferation. Splenocytes were stained with 5 μM carboxyfluorescein succinimidyl ester (CFSE) and seeded in triplicate in 24-well culture plates at a density of 2 x 106 cells/mL in RPMI-1640 medium supplemented with 10%fetal bovine serum with or without 100 μg/mL OVA. 5 μg/mL concanavalin A (ConA) was used as positive controls. After 48 hours, cells were harvested and stained with phycoerythrin (PE)-conjugated anti-CD4+ Ab or PE-conjugated anti-CD8+ Ab and analyzed by FACScan flow cytometer. The figures are showing the results of one independent experiment. N=4 in each group. Bars indicate the mean ± S.D. SAL, saline; OVA, ovalbumin. *p<0.05 vs SAL.

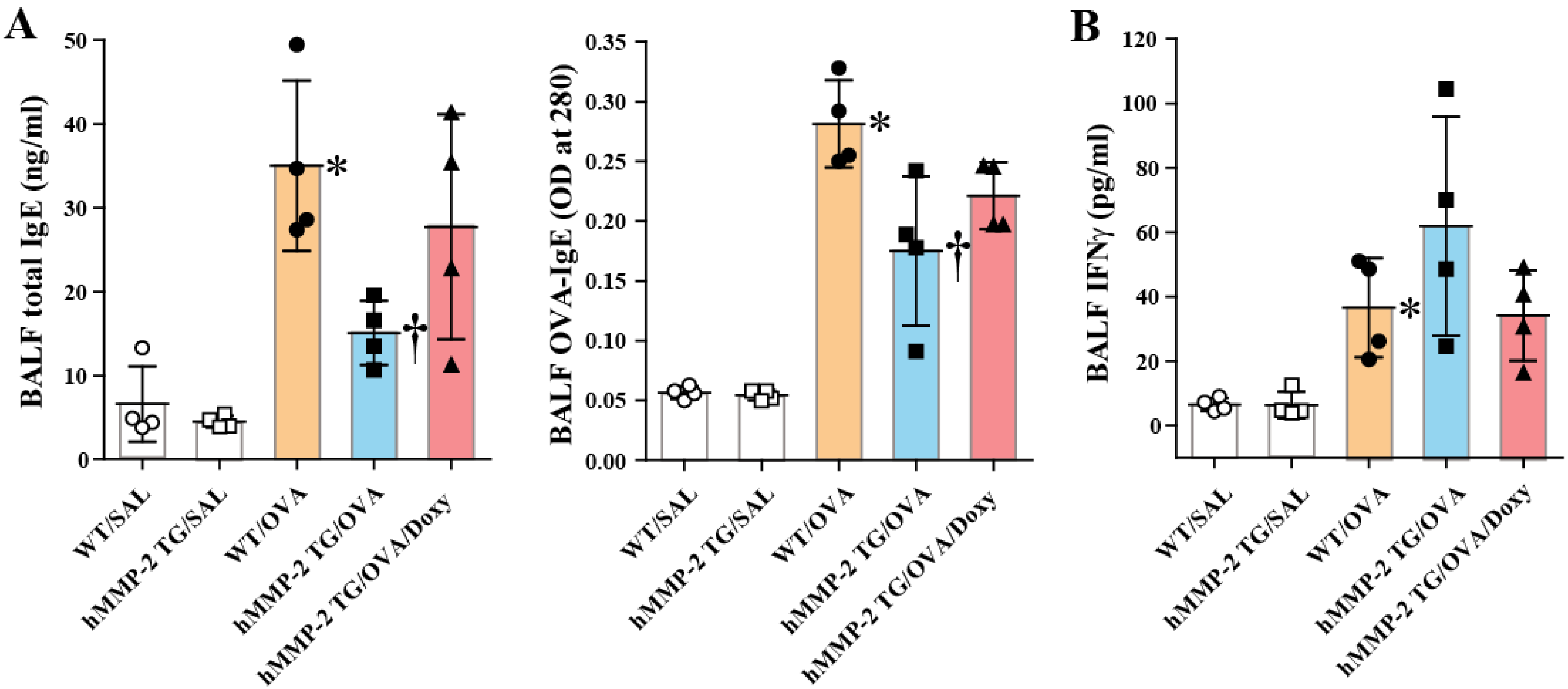

Supplementary Fig. 11. An inhibitor of matrix metalloproteinase (hMMP) tends to block the beneficial effect of hMMP-2 on allergic asthma. Wild type (WT) mice and hMMP-2 transgenic (TG) mice received intraperitoneal injection of ovalbumin (OVA) or saline (SAL) and then challenged with OVA or SAL as described under materials and methods. A group of hMMP-2 TG mice sensitized and challenged with OVA was fed with baits containing doxycycline (Doxy) 2 weeks before sensitization. Samples were collected and immunoglobulins and cytokines were measured by enzyme immune assays. The figures are showing the results of one independent experiment. N=4 in each group. Bars indicate the mean ± S.D. WT, wild type; SAL, saline; TG, transgenic; OVA, ovalbumin. *$p<0.05$ vs WT/SAL; †$p<0.05$ vs WT/OVA

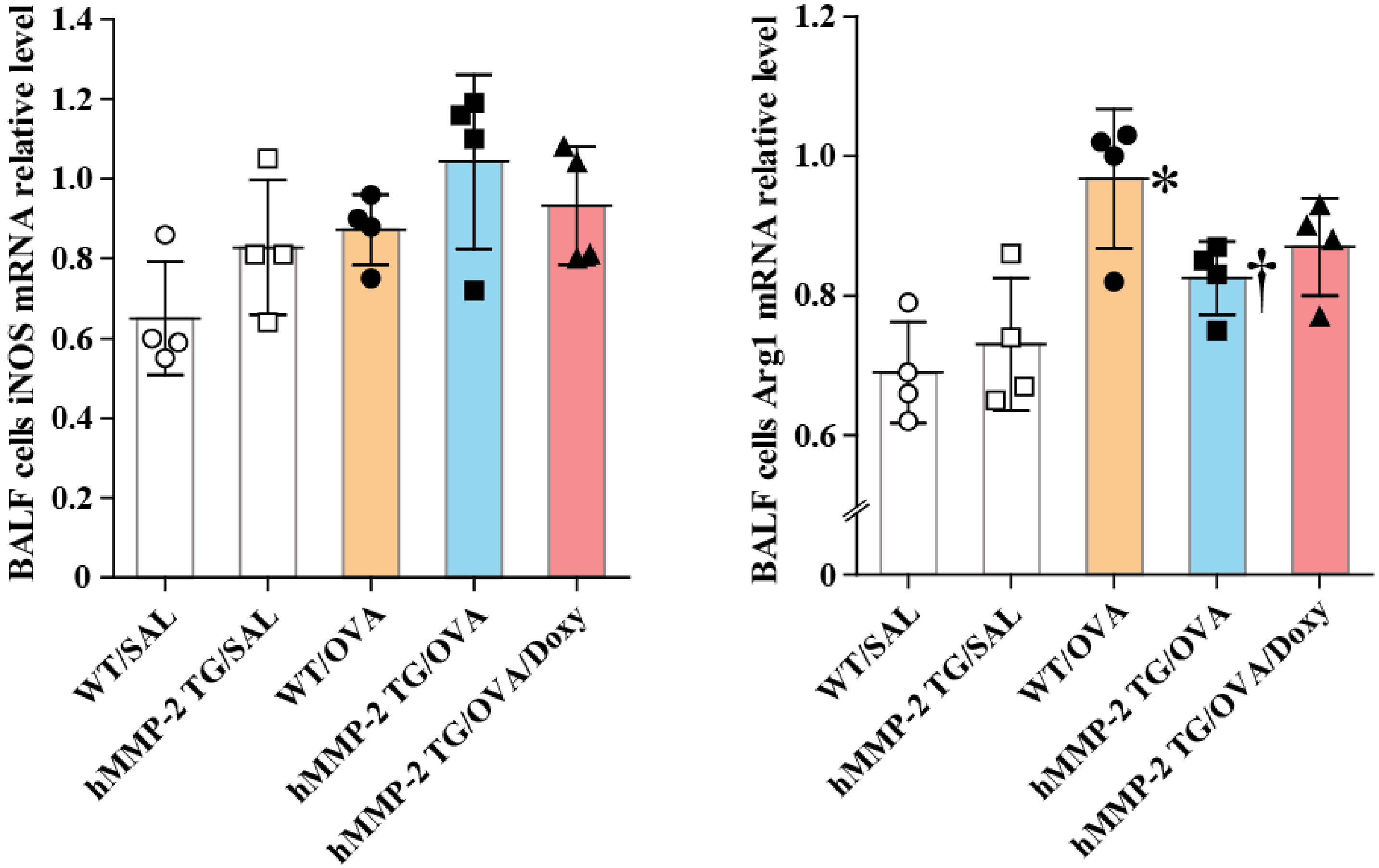

Supplementary Fig. 12. Increased markers of M1 polarization in BALF cells from hMMP-2 TG mice. Wild type (WT) and proMMP-2 TG became allergic after sensitization and challenge with ovalbumin (OVA). A group of hMMP-2 TG mice was fed with baits containing 5% doxycycline before, during sensitization and challenge with OVA. Mice receiving saline were the controls. The relative mRNA expression of inducible nitric oxide synthase (iNOS) and arginase 1 (Arg1) was evaluated by reverse-transcriptase polymerase chain reaction. The figures are showing the results of one independent experiment. N=4 in each group. Bars indicate the means ± S.D. Statistical difference was evaluated by analysis of variance with Tukey test. hMMP-2, human matrix metalloproteinase-2. *p<0.05 vs WT/SAL; †p<0.05 vs WT/OVA.

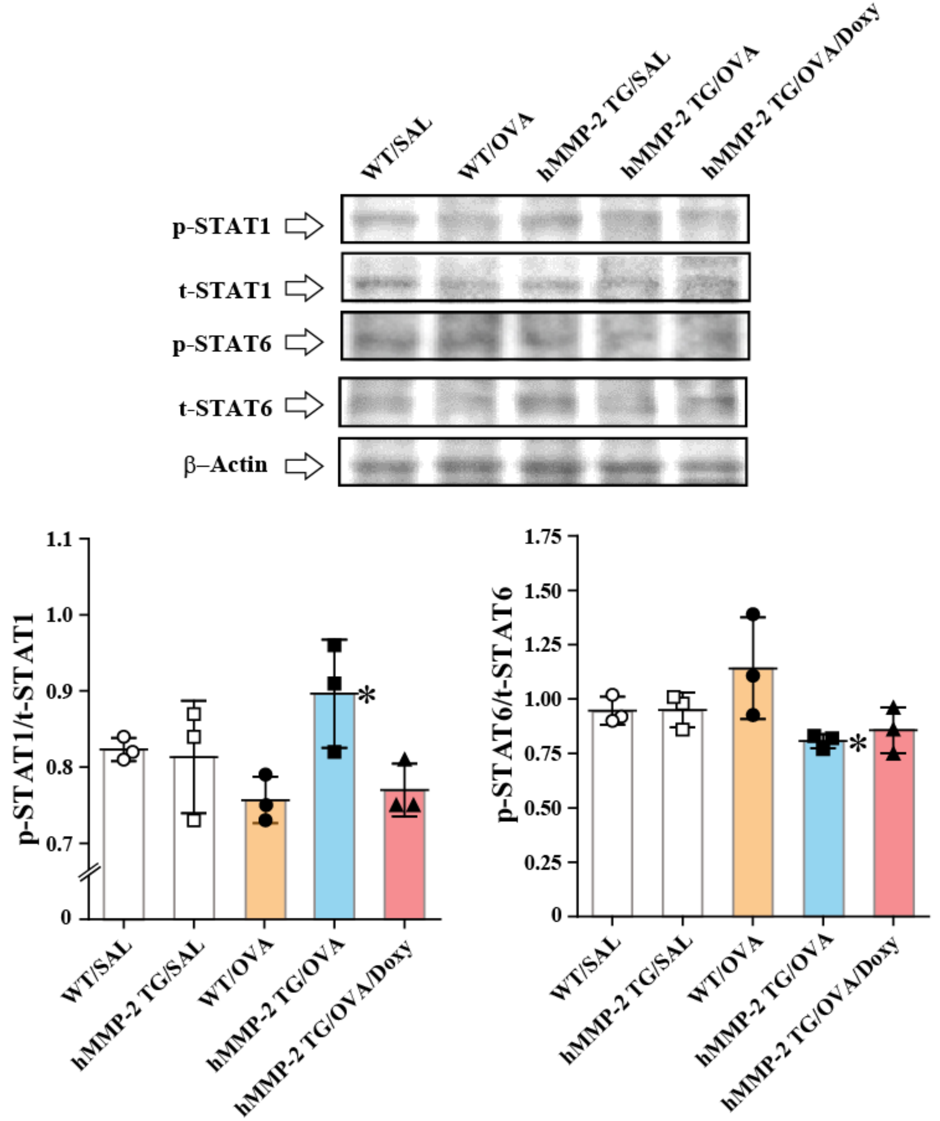

Supplementary Fig. 13. Increased activation of STAT1 but reduced activation of STAT6 in lung tissue from human MMP-2 TG mice sensitized and challenged with allergen. Wild type (WT) mice and hMMP-2 transgenic (TG) mice received intraperitoneal injection of ovalbumin (OVA) or saline (SAL) and then challenged with OVA or SAL as described under materials and methods. The lung tissue of each group (n=3) group of muse was homogenized, the concentration of protein was measured and then equal amount of protein was used to evaluate phosphorylation of STAT1 and STAT6 by Western blotting. The figures are showing the results of one independent experiment. N=4 in each group. Bars indicate the means ± S.D. Statistical difference was evaluated by analysis of variance with Tukey test. hMMP-2, human matrix metalloproteinase-2. *p<0.05 vs WT/OVA.

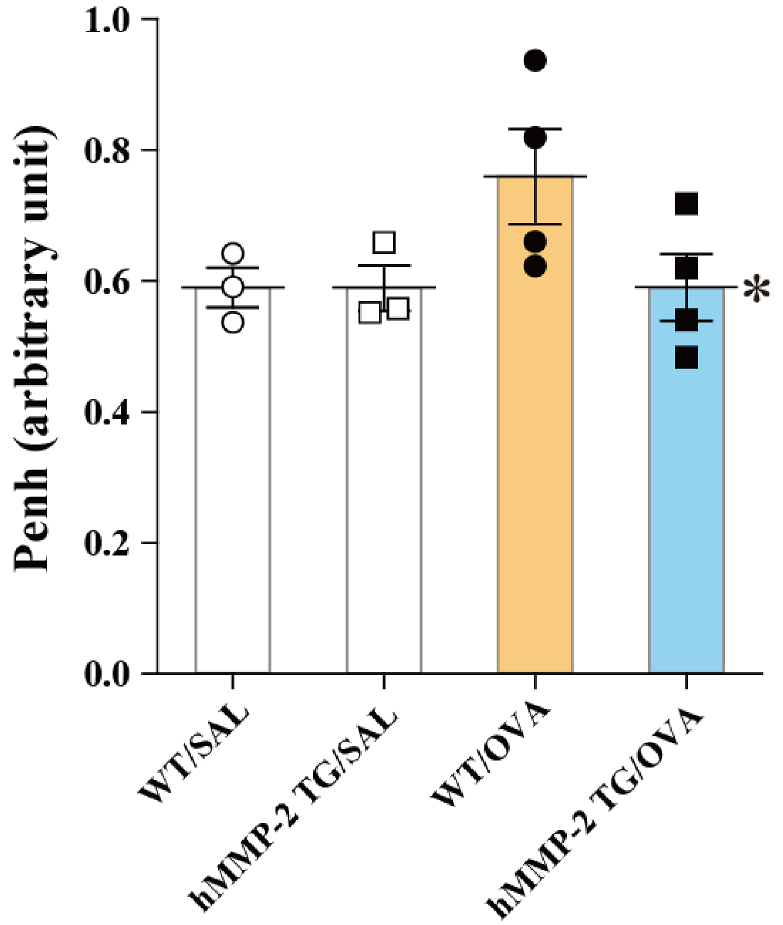

Supplementary Fig. 14. Decreased airway hyperresponsiveness in allergen-sensitized and challenged hMMP-2 TG mice compared to WT counterparts before using all mice for *in vivo* evaluation of macrophage polarization and T cell population. Wild type (WT) and proMMP-2 TG became allergic after sensitization and challenge with ovalbumin (OVA). Mice receiving saline were the controls. Penh was measured using a plethysmography after inhalation of 10 mg/ml of methacholine. Statistical difference was evaluated by analysis of variance with Tukey test. Bars indicate the means ± S.E.M. hMMP-2, human matrix metalloproteinase-2; WT, wild type; SAL, saline; TG, transgenic; OVA, ovalbumin. WT/SAL with n=3, hMMP-2 TG/SAL n=3, WT/OVA n=4, hMMP-2 TG/OVA n=4. *$p<0.05$ vs WT/OVA.

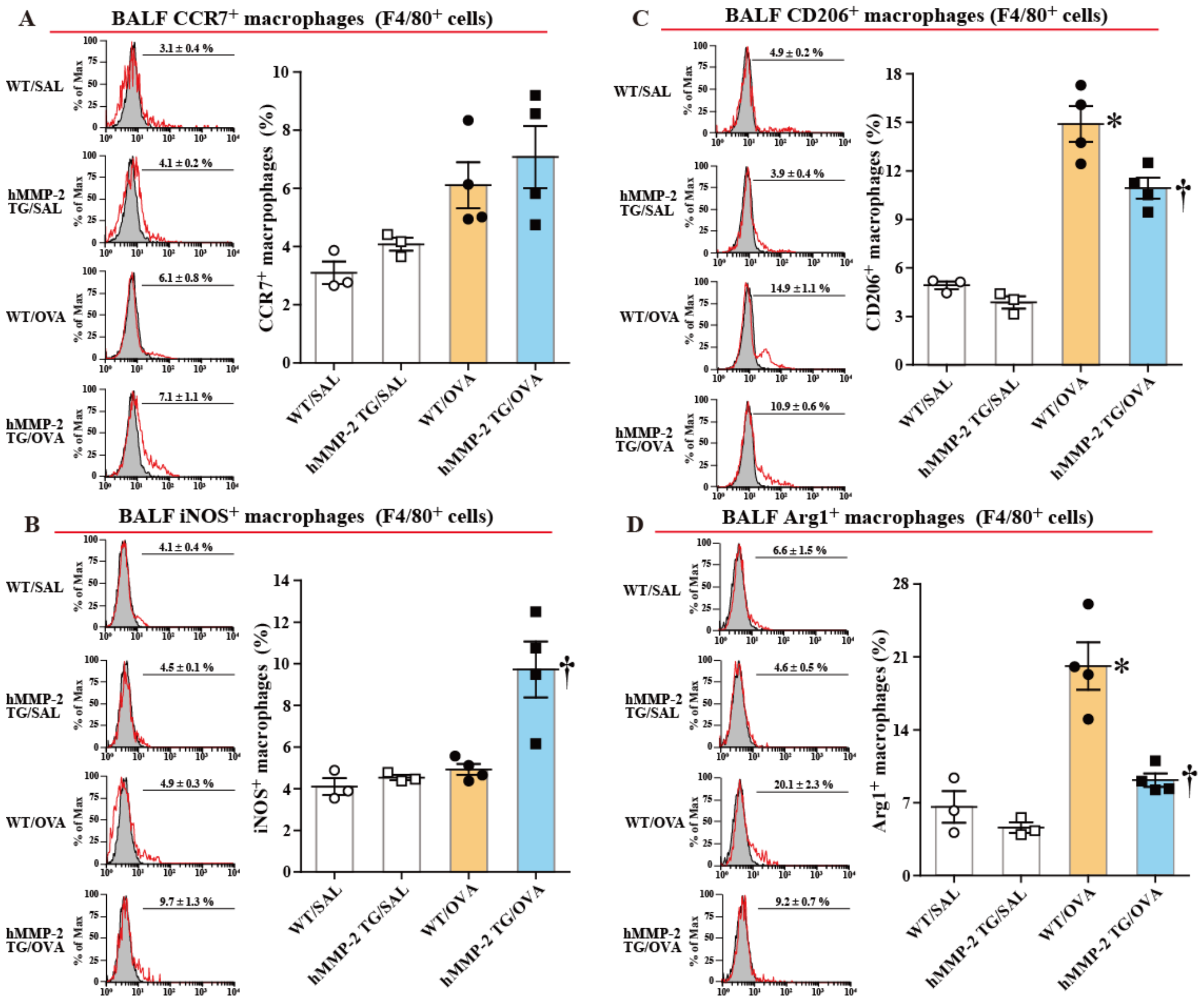

Supplementary Fig. 15. Increased percentage of M1 macrophages in bronchoalveolar lavage fluid from hMMP-2 transgenic mice. Wild type (WT) and proMMP-2 TG became allergic after sensitization and challenge with ovalbumin (OVA). Mice receiving saline were the controls. The percentage of CCR7+ (A), iNOS (B), CD206+ (C) and Arg1 (D) was evaluated by flow cytometry after gating F4/80+ cells. Bars indicate the means ± S.E.M. Statistical difference was evaluated by analysis of variance with Tukey test. hMMP-2, human matrix metalloproteinase-2. WT/SAL with n=3, hMMP-2 TG/SAL n=3, WT/OVA n=4, hMMP-2 TG/OVA n=4. *p<0.05 vs WT/SAL; †p<0.05 vs WT/OVA.

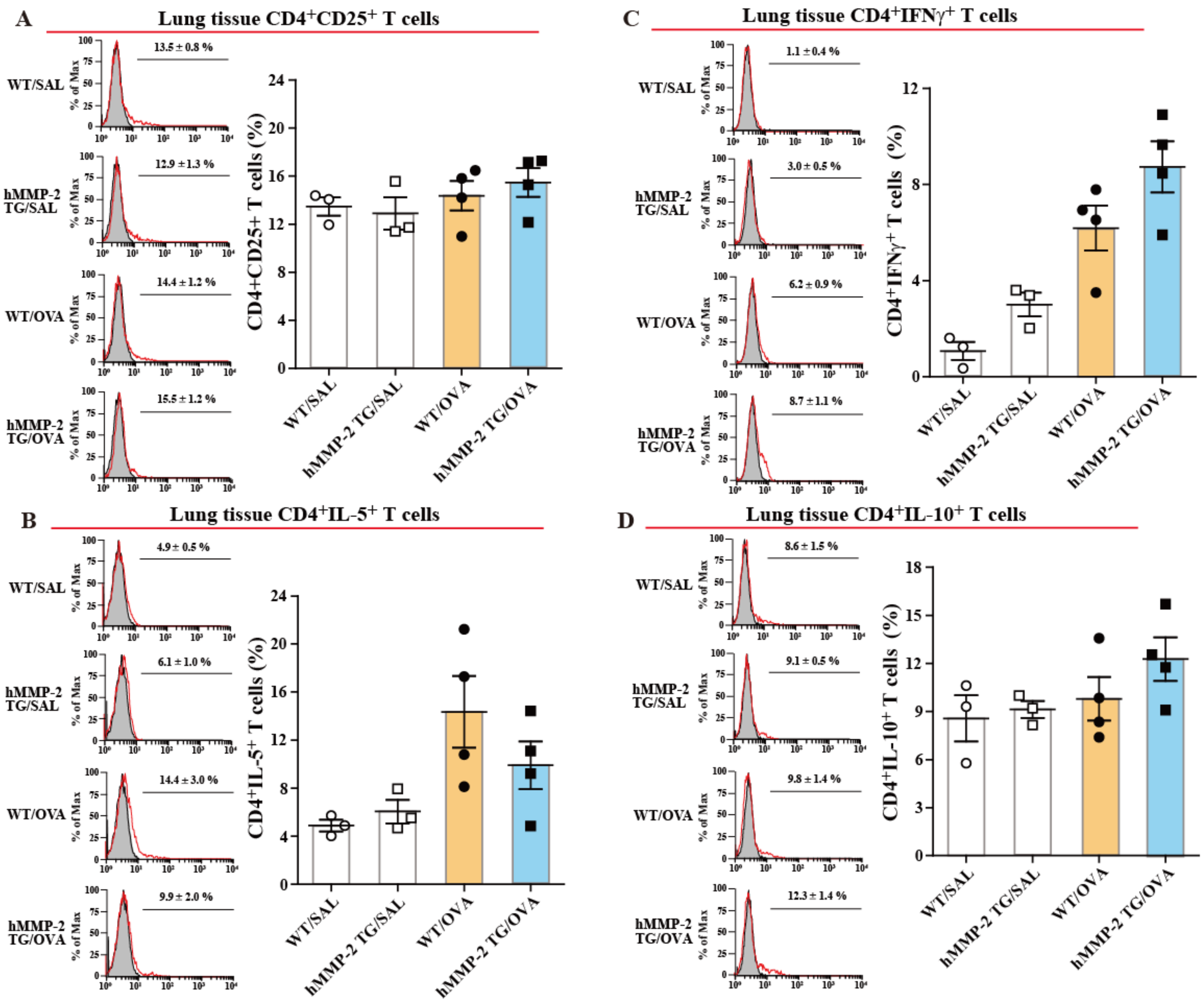

Supplementary Fig. 16. The percentage of CD4+CD25+ T cells remains unchanged in lung tissue from hMMP-2 transgenic mice. Wild type (WT) and proMMP-2 TG became allergic after sensitization and challenge with ovalbumin (OVA). Mice receiving saline were the controls. The percentage of CD4+CD25+ (A), CD+IFNγ+ (B), CD4+IL-5 + (C) and CD4IL-10+ (D) T cells was evaluated by flow cytometry. Bars indicate the means ± S.E.M. Statistical difference was evaluated by analysis of variance with Tukey test. hMMP-2, human matrix metalloproteinase-2. WT/SAL with n=3, hMMP-2 TG/SAL n=3, WT/OVA n=4, hMMP-2 TG/OVA n=4.

Supplementary Table 1. Patients characteristics

Variables	Mean values and No of patients
No of patients	27
Sex M/F	12/15
Age (years-old)	62.2 ± 17.3
FEV1 (L)	2.1 ± 1.0
FEV1/FVC (%)	72.6 ± 12.2
FEV1/pFEV1 (%)	87.1 ± 24.6
Blood eosinophils (%)	5.3 ± 4.0
Serum IgE (U/ml)	415.0 ± 701.1
Asthma severity according to GINA	
2	4
3	17
4	6
Allergens	
Wheat	2
Cedar	11
House dust	7
Acarus	6
Ragweed	1

Data are the mean ± S.D. FEV1, forced expiratory volume in one second; FVC, forced volume vital capacity; pFEV1, predicted forced expiratory volume in one second; ICS, inhaled corticosteroids; LABA, long-acting beta-agonists; LTRA, leukotriene receptor antagonists.

Supplementary Table 2. Primers for RT-PCR

	Sequence (5' -> 3')	Tm	Reference	Location	Size
GAPDH					
Sense	GGAGCGAGATCCCTCCAAAAT	61.6	NM_001256799	108-128	197 bp
Antisense	GGCTGTTGTCATACTTCTCATGG	60.9		304-282	
IL-5					
Sense	TCTACTCATCGAACTCTGCTGA	60.0	NM_000879	112-133	132 bp
Antisense	CCCTTGCACAGTTTGACTCTC	60.8		243-223	
IL-13					
Sense	CCTCATGGCGCTTTTGTTGAC	62.4	NM_002188	39-59	134 bp
Antisense	TCTGGTTCTGGGTGATGTTGA	60.7		172-152	
MCP-1					
Sense	CTCTGCCGCCCTTCTGTGCCTG	74.0	NM_002982	82-103	539 bp
Antisense	ACATCCCAGGGGTAGAACTCTGG	72.0		620-602	
IL-10					
Sense	TCAAACAAAGGACCAGCTGGACAACATACTG	90.0	NM_010548	232-262	421 bp
Antisense	CTGTCTAGGTCCTGGAGTCCAGCAGACTCA	72.0		652-623	

GAPDH: glyceraldehyde-3-phosphate dehydrogenase; IL: interleukin

Supplementary Table 3. Secretion of cytokines and immunoglobulin induced by ovalbumin in spleen cells

	IFNγ (pg/ml)		IL-2 (pg/ml)		IL-4 (pg/ml)		IL-5 (pg/ml)		IgE (ng/ml)	
	SAL	OVA	SAL	OVA	SAL	OVA	SAL	OVA	SAL	OVA
WT/SAL	32.9 ± 6.2	59.9 ± 9.5	28.2 ± 2.5	]29.2 ± 7.8*	20.3 ± 5.3	17.8 ± 5.4*	19.0 ± 8.6	19.6 ± 3.7*	1.2 ± 0.1	1.2 ± 0.1*
hMMP-2 TG/SAL	29.7 ± 13.8	54.6 ± 7.3†	23.6 ± 2.3	26.9 ± 1.6†	20.1 ± 5.2	24.4 ± 10.0	17.4 ± 3.4	25.9 ± 6.8	1.1 ± 0.1	1.3 ± 0.1
WT/OVA	26.5 ± 7.6	425.9 ± 25.6‡	20.5 ± 1.2	132.7 ± 40.0‡	42.2 ± 2.8	79.0 ± 39.8‡	134.5 ± 42.0	324.5 ± 87.7†	11.5 ± 5.1	29.0 ± 26.4
hMMP-2 TG/OVA	21.3 ± 10.7	1000.7 ± 600.7¶	21.0 ± 3.6	300.7 ± 123.8¶	23.5 ± 6.9	36.5 ± 7.6¶	73.6 ± 13.5	291.2 ± 16.2¶	8.6 ± 1.2	14.1 ± 1.4¶

Data are the mean ± S.D. The table is showing the results of one independent experiment. N=4 in each group. IFNγ, interferonγ; IL-2, interleukin-2; IL-4, interleukin-4; IL-5, interleukin-5; SAL, saline; hMMP-2 TG, human matrix metalloproteinases, WT, wild type; OVA, ovalbumin. *$p<0.05$ vs WT/OVA; †$p<0.05$ vs hMMP-2 TG/OVA; ‡$p<0.05$ vs hMMP-2 TG/OVA; ¶$p<0.05$ vs hMMP-2 TG/OVA + SAL
